# Supplementary material for: A DNA methylation atlas of normal human cell types
Source: Nature. 2023 Jan 4;613(7943):355–64. doi: 10.1038/s41586-022-05580-6 (PMC9811898; doi:10.1038/s41586-022-05580-6)
Supplement: Supplementary file 1 — Legends for Supplementary Tables; cell sorting: FACS plots and RNA/methylation enrichments; genome-wide segmentation to methylation blocks; selection of type-specific markers; UXM fragment-level deconvolution algorithm; purity estimations: proportion of unmethylated fragments in various samples; analysis of cfDNA composition in patients with COVID-19; dissociation protocols; appendix: RBC lysis buffer (X10); Supplementary Table 18: antibodies for flow cytometry; and Supplementary Table 19: reagents used. [file 41586_2022_5580_MOESM1_ESM.pdf]

nature portfolio

https://doi.org/10.1038/s41586-022-05580-6

Supplementary information

A DNA methylation atlas of normal human cell types

In the format provided by the authors and unedited

Supplemental Information
A DNA methylation atlas of normal human cell types

Netanel Loyfer[1,22], Judith Magenheim[2,22], Ayelet Peretz[2,22], Gordon Cann[3], Joerg Bredno[3], Agnes Klochendler[2], Ilana Fox-Fisher[2], Sapir Shabi-Porat[1], Merav Hecht[2], Tsuria Pelet[2], Joshua Moss[2,4], Zeina Drawshy[2], Hamed Amini[3], Patriss Moradi[3], Sudharani Nagaraju[3], Dvora Bauman[5] , David Shveiky[5], Shay Porat[5], Uri Dior[5], Gurion Rivkin[6], Omer Or[6], Nir Hirshoren[7], Einat Carmon[8,20], Alon Pikarsky[9], Abed Khalaileh[8], Gideon Zamir[8], Ronit Grinbaum[8], Machmud Abu Gazala[8], Ido Mizrahi[8], Noam Shussman[8], Amit Korach[10], Ori Wald[10], Uzi Izhar[10], Eldad Erez[10], Vladimir Yutkin[11], Yaacov Samet[12], Devorah Rotnemer Golinkin[13], Kirsty L. Spalding[14], Henrik Druid[15,16], Peter Arner[17], A.M. James Shapiro[18], Markus Grompe[19], Alex Aravanis[3,21], Oliver Venn[3], Arash Jamshidi[3], Ruth Shemer[2], Yuval Dor[2,*], Benjamin Glaser[13,*], and Tommy Kaplan[1,2,*]

1. School of Computer Science and Engineering, The Hebrew University of Jerusalem, Israel
2. Dept. of Developmental Biology and Cancer Research, Institute for Medical Research Israel-Canada, Hadassah Medical Center and Faculty of Medicine, Hebrew University of Jerusalem, Israel
3. GRAIL, Inc., Menlo Park, California, United States of America
4. Sharett Institute of Oncology, Hadassah Hebrew University Medical Center, Jerusalem, Israel
5. Dept. of Obstetrics and Gynecology, Hadassah Medical Center and Faculty of Medicine, Hebrew University of Jerusalem, Israel
6. Dept. of Orthopedics, Hadassah Medical Center and Faculty of Medicine, Hebrew University of Jerusalem, Israel
7. Dept. of Otolaryngology, Hadassah Medical Center and Faculty of Medicine, Hebrew University of Jerusalem, Israel
8. Dept. of General Surgery, Hadassah Medical Center and Faculty of Medicine, Hebrew University of Jerusalem, Israel
9. Surgery Division, Hadassah Medical Center and Faculty of Medicine, Hebrew University of Jerusalem, Israel
10. Dept. of Cardiothoracic Surgery, Hadassah Medical Center and Faculty of Medicine, Hebrew University of Jerusalem, Israel
11. Dept. of Urology, Hadassah Medical Center and Faculty of Medicine, Hebrew University of Jerusalem, Israel
12. Dept. of Vascular Surgery, Shaare Zedek Medical Center, Jerusalem Israel
13. Dept. of Endocrinology and Metabolism, Hadassah Medical Center and Faculty of Medicine, Hebrew University of Jerusalem, Israel
14. Dept. of Cell and Molecular Biology, Karolinska Institutet, Stockholm, Sweden
15. Dept. of Oncology-Pathology, Karolinska Institutet, Stockholm, Sweden
16. Dept. of Forensic Medicine, The National Board of Forensic Medicine, Stockholm, Sweden
17. Dept. of Medicine (H7) and Karolinska University Hospital, Karolinska Institutet, Stockholm, Sweden
18. Dept. of Surgery and the Clinical Islet Transplant Program, University of Alberta, Edmonton, Canada
19. Papé Family Pediatric Research Institute, Oregon Health & Science University, Portland, OR, USA
20. Current address: Department of Surgery, Samson Assuta Ashdod University Hospital
21. Current address: Illumina, Inc., San Diego, California, United States of America
22. These authors contributed equally

* Corresponding authors

Table of Contents

Legends for Supplementary Tables 3

Cell sorting – FACS plots and RNA/methylation enrichments 5

Genome-wide segmentation to methylation blocks 21

Selection of type-specific markers 24

UXM fragment-level deconvolution algorithm 25

Purity estimations: proportion of unmethylated fragments in various samples 29

Analysis of cfDNA composition in COVID-19 patients 31

Dissociation Protocols 32

- Adipocytes 32
- Bladder epithelium 32
- Blood Granulocytes 32
- Breast basal and luminal epithelium 33
- Cardiomyocytes (Nuclei) 34
- Colon epithelium, Small intestine epithelium, Gall-bladder epithelium, Colon macrophages 35
- Cortical Neurons 36
- Endometrial epithelium 36
- Endothelium from Vein 37
- Erythrocyte progenitors 38
- Esophagus epithelium 38
- Fallopian Tube epithelium 38
- Fibroblasts 39
- Gastric antrum, body or fundus epithelium 40
- Hepatocytes 41
- Kidney Tubular and glomerular epithelium 42
- Larynx and Pharynx epithelium 42
- Lung alveolar epithelium, bronchial epithelium, interstitial lung macrophages, lung alveolar macrophages. 43
- Mononuclear Cells (Lymphocytes and Monocytes) 44
- Ovary epithelium 44
- Pancreas islet/duct/acinar cells 45
- Prostate epithelium 45
- Thyroid epithelium 46
- Tongue epithelium 47
- Tonsil epithelium 47

Appendix - RBC lysis buffer (X10): 48

Supplementary Table S18 - Antibodies for Flow Cytometry 49

Supplementary Table S19 - Reagents used 51

Legends for Supplementary Tables

Supplementary Table S1. List of 205 samples, including tissue of origin, cell type, cell type group, germinal layer of cell type, source of tissue sample, clinical details of donor, sorting details, and sequencing depth. Abbreviations: Had-HU = Hadassah Hospital/Hebrew University; KI = Karolinska Institute; IIDP = Integrated Islet Distribution Program; OHSU = Oregon Health & Science University; UA = University of Alberta

Supplementary Table S2. List of methylation blocks with ≥4 CpGs that show the highest variability (top 1%) across samples.

Supplementary Table S3. List of 39 cell types (top) and 12 super groups (bottom).

Supplementary Table S4. (A) List of 953 cell type-specific unmethylated markers. Also included are additional 293 markers, uniquely unmethylated in combinations of a few related cell types (e.g. pancreatic alpha, beta, and delta cells, gastrointestinal cell types, etc.). (B) Set of top 250 unmethylated markers, per cell type (11,713 regions). (C) Set of top 1,000 unmethylated markers, per cell type (50,287 regions).

Supplementary Table S5. Cell type-specific markers are enriched for functional terms. Shown are all enriched terms for each marker group, with a significance threshold of FDR≤1E-10.

Supplementary Table S6. Transcription factor binding site analysis (using HOMER). (A) Enriched motifs for top 1,000 uniquely unmethylated regions for each cell type. (B) Enriched motifs for unmethylated regions per cell type. (C) Enriched motifs for unmethylated regions that overlap H3K27ac, but not H3K4me3, ChIP-seq peaks, for each cell type. (D) Roadmap and ENCODE accession numbers for ATAC-seq and ChIP-seq peaks, and chromHMM annotations. Shown are HOMER's findMotifsGenome.pl outputs, including motif name, binomial p-value, log p-value, and q-value and percent and number of sequences containing the motif.

Supplementary Table S7. Methylation marker-gene associations. Each cell type-specific unmethylated region was associated with over-expressed genes (up to 750Kb) in related tissues.

Supplementary Table S8. Fragment-level deconvolution of 23 blood and 23 plasma samples.

Supplementary Table S9. Fragment-level deconvolution of 104 WGBS samples from Roadmap and ENCODE.

Supplementary Table S10. Fragment-level deconvolution of 205 atlas samples.

Supplementary Table S11. Fragment-level deconvolution of atlas samples, using leave-one-out cross-validation, for cell types with ≥3 replicates.

Supplementary Table S12. List of cell type-specific unmethylated markers (top 25), which overlap Illumina 450K BeadChip array.

Supplementary Table S13. List of cell type-specific unmethylated markers (top 25), which overlap Illumina EPIC BeadChip array.

Supplementary Table S14. List of cell type-specific unmethylated markers (top 25), covered by RRBS regions.

Supplementary Table S15. List of cell type-specific unmethylated markers (top 25), covered by Illumina TruSeq EPIC hybrid capture panel.

Supplementary Table S16. List of cell type-specific unmethylated markers (top 25), covered by Roche SeqCapEpi hybrid capture panel.

Supplementary Table S17. List of cell type-specific unmethylated markers (top 25), covered by Agilent SureSelectXT hybrid capture panel.

Supplementary Table S18. Antibodies for Flow Cytometry

Supplementary Table S19. Reagents used

Supplementary Dataset 1. Genome-wide set of unmethylated regions per cell type, annotated. Zip file contains 39 bed files, each with all genomic regions (blocks of 4 CpGs or more, where ≥85% of sequenced fragments are unmethylated in ≥85% of covered CpGs). Bed file information contain position (chr, from, to, CpG_from, CpG_to, hg19), regional annotation by HOMER and neighboring gene, and annotations of where the regions is ≤1Kb from a TSS, overlapping a CpG island, chromHMM annotation in that cell type or similar, ChIP-seq peak annotations for H3K4me3 (promoter), H3K27ac (gene regulation), H3K4me1 (enhancer), H3K27me3, overlapping ATAC-seq peak, presence of CTCF binding site, and CTCF ChIP-seq peak.

Supplementary Dataset 2. Genome-wide set of unmethylated regions per cell type, overlapping H3K27ac, but not J3K4me3, ChIP-seq peaks, annotated. Zip file containing bed files, listing for each cell type a set of putative enhancers, defined as the subset of hypo-methylated regions (Extended Dataset 1), that are marked by active gene regulation chromatin marks (H3K27ac peaks), but lack promoter marks (H3K4me3 peaks).

Cell sorting – FACS plots and RNA/methylation enrichments

Fresh tissue was obtained at surgery and dissociated (optimized per tissue type), then incubated with antibodies, and FACS-sorted. Sorted cells were analyzed using mRNA analysis for key cell type-specific genes, or using targeted PCR for cell type-specific DNA methylation markers. Finally, DNA methylation was analyzed using whole-genome bisulfite sequencing:

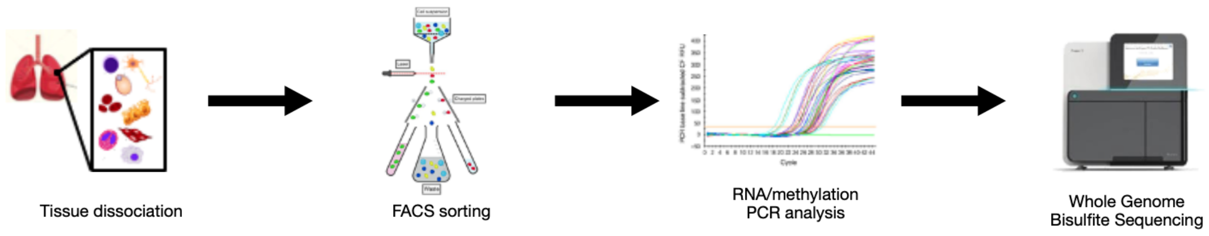

Below we present FACS plots for cell sorting, isotype controls and gating strategy used for each cell type. For example, epithelial cells were sorted from lineage-negative population (CD31-, CD45- and CD235a-) using EpCam staining.

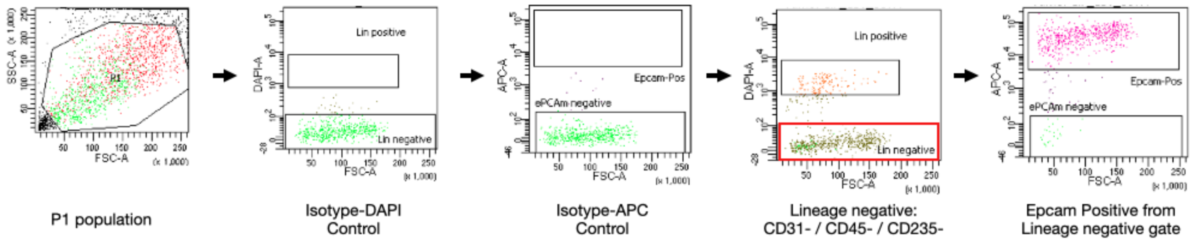

For each cell type, FACS plots of sorted cells using immunostaining for different antibodies according to the target sorted cells are shown on the left. On the right, we present relative enrichment in RT-qPCR (expression of cell type-specific marker genes), or the cumulative percentage of unmethylated fragments in cell type-specific methylation markers. These are compared to the expression or methylation in the tissue.

Validations of cell populations were done using two alternative methods of marker gene mRNA expression or DNA methylation using known markers.

RNA extraction and Quantitative Real-Time PCR. Total RNA from 5000 cells (of the positive population) and 1mm of whole tissue were extracted with RNeasy Micro kit (QIAGEN) and reverse transcription was performed with a qScript kit (Quanta Biosciences) according to the manufacturer's instructions. Quantitative real-time PCR (qRT-PCR) was performed on a CFX96 Real-Time System (Bio-Rad) with PerfeCTa SYBR Green SuperMix (Quanta Biosciences) and gene-specific primers:

Forward	Reverse	PRIMRES
cctttgccgatccgccg	gatatcatcatccatggtgagctgg	**Actin**
gtatgagaaggctgagataaag	cttcaaagatgtcttcgtcc	**EpCAM**
CGCAATAGACAAGGACATAAC	TATCGTGATTATCCGTGAGG	**ve-cad (CDH5)**
atttacaggggacatcctac	ccattgatatccagaaagtcc	**ATP6V1B1**
cttttcatgagatcgtgacc	gactgcttaggagaagagag	**NPHS2**
tccctggatctatttgtgatg	ccttctgttgatatctctcttg	**PLAC8**

gaacaagctcctgaagatac	atacatgccttcaaacacag	**PAPA2**
gatagacaccaatgagattgc	tttgaagaaaaagagctcgc	**MMP11**
ctatcacctcctaaaggacc	ttgtgtcaaacttgctgtag	**CSH1**
CCGAGAGCTACACGTTC	TCTTCAAAATTCACTCTGCC	**CDH1- (E-cad)**
GAATGACTCCAACTACATTGTC	GTGTAGACACAGTCAAAGATG	**CSF1R**
TCAAGTTCCTTCATCCATTC	CATCCACTCAATATCAGGAAG	**PDGFRA- (CD140a)**
CATATGACTATAACAGAGTGCC	ATGTATTTGCTTGTTCCTC	**PTPRC (CD45)**

Targeted-PCR DNA methylation biomarkers. DNA was extracted using DNeasy Blood & Tissue (QIAGEN) and treated with bisulfite (Zymo). We used a multiplex 2-step PCR protocol as fully described in Neiman et al. (PMID 32573495).

Targeted methylation primers as previously published for liver markers (Lehmann-Werman et al., PMID PMC4822610; Lehmann-Werman et al., PMID 29925683), and for cardiomyocyte markers (Zemmour et al., PMID 29691397; Pollak et al., PMID 35978264). Other markers include (all coordinates are hg19): Cardio1: chr2:86255694, Cardio2: chr18:55922851, Cardio3: chr18:55923678, Alpha2: chr13:28492600, Alpha3: chr14:56805397, Acinar 1: chr10:133806781, Acinar2: chr1:15783130, Acinar3: chr7:50800912, Acinar4: chr3:48413633, Acinar5: chr10:112618839, Beta2: chr16:30958103, Beta3: chr10:79601918, Delta1: chr7:129945588, and Delta2: chr04:6986661.

	FACS	RNA / Methylation analysis
Cardiomyocytes	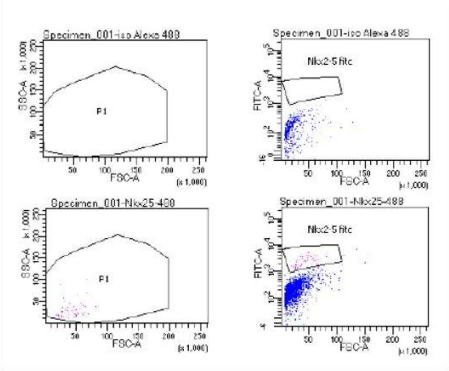	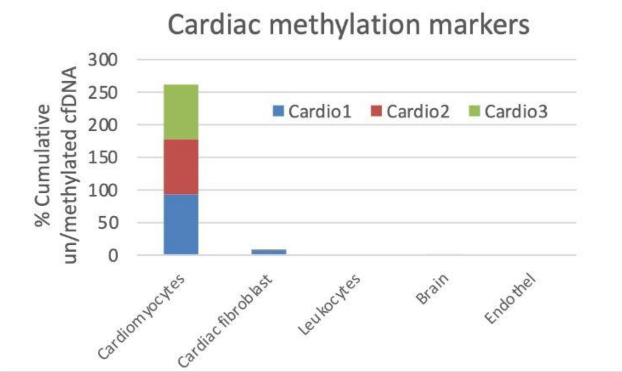
Liver- Hepatocytes	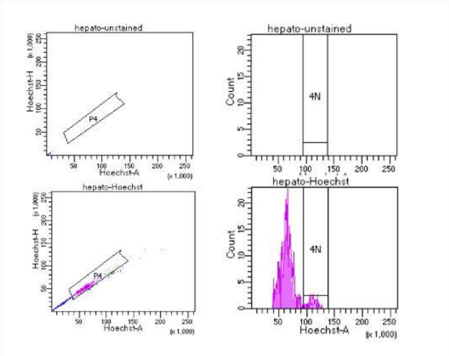	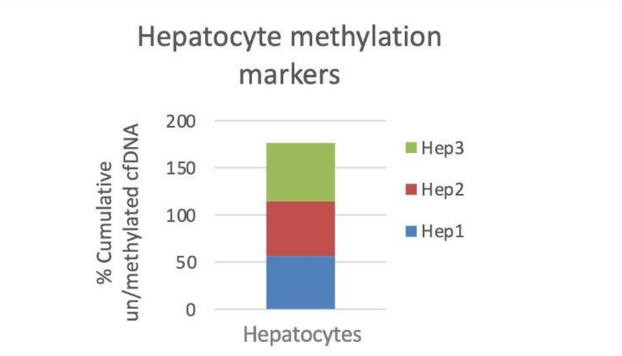
Thyroid- Epithelium	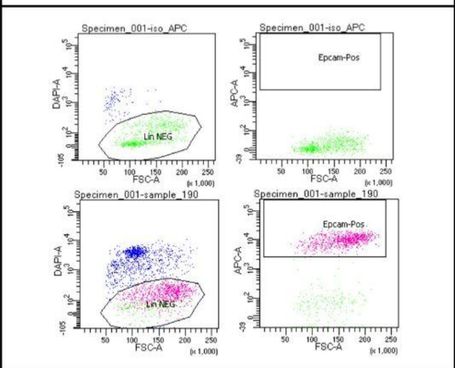	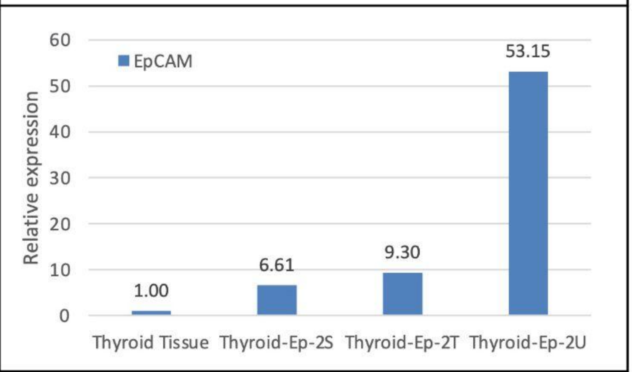
Fallopian Tube- Epithelium	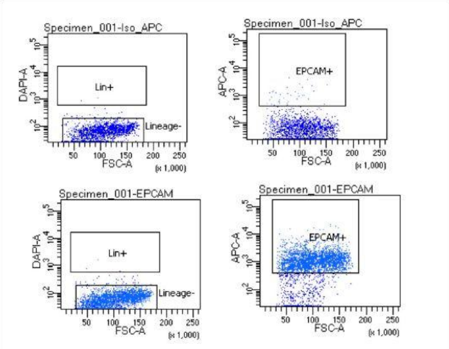	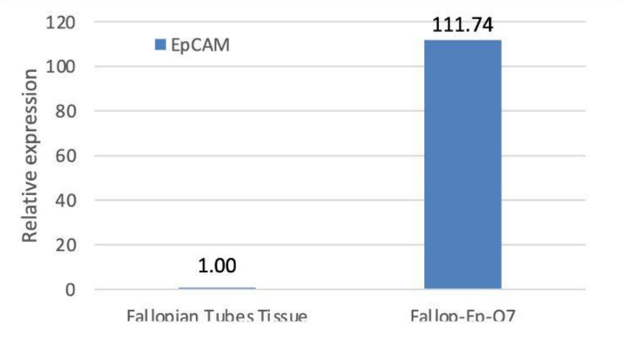

<table>
<tr><th></th><th>FACS</th><th>RNA / Methylation analysis</th></tr>
<tr><td>Ovary-Epithelium</td><td>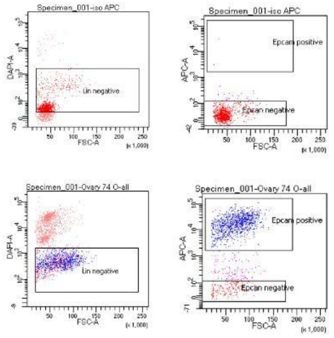
</td><td>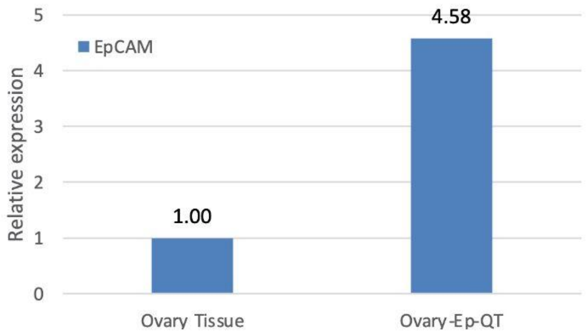
</td></tr>
<tr><td>Endometrium-Epithelium</td><td>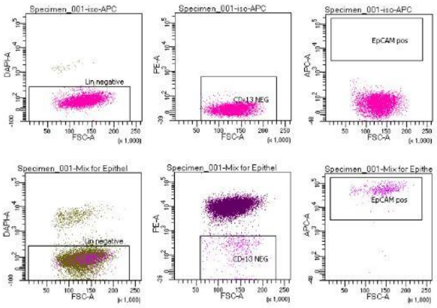
</td><td>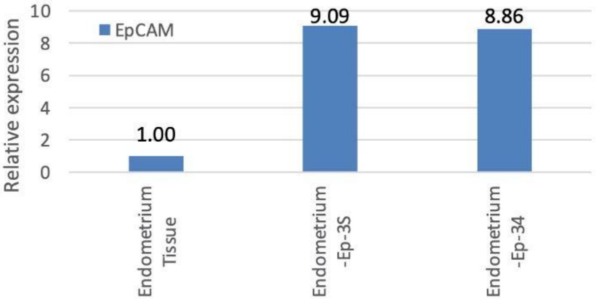
</td></tr>
<tr><td>Tonsil palatine-Epithelium</td><td>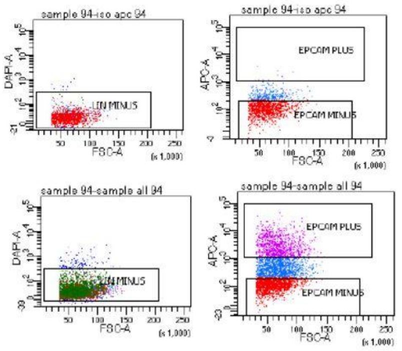
</td><td>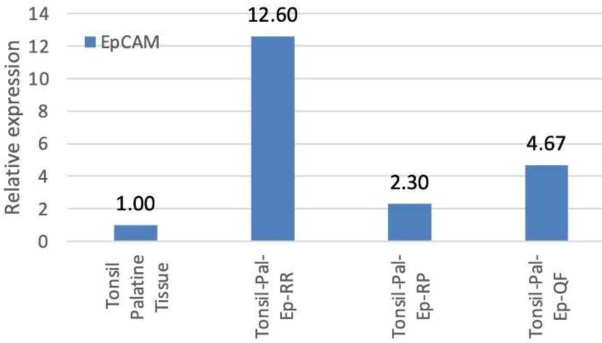
</td></tr>
<tr><td>Tonsil pharyngeal-Epithelium</td><td>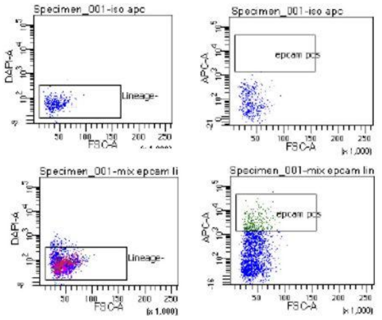
</td><td>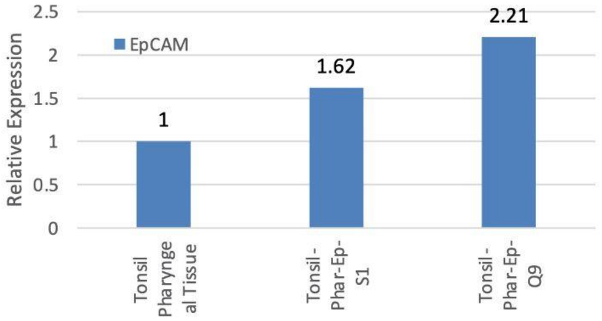
</td></tr>
</table>

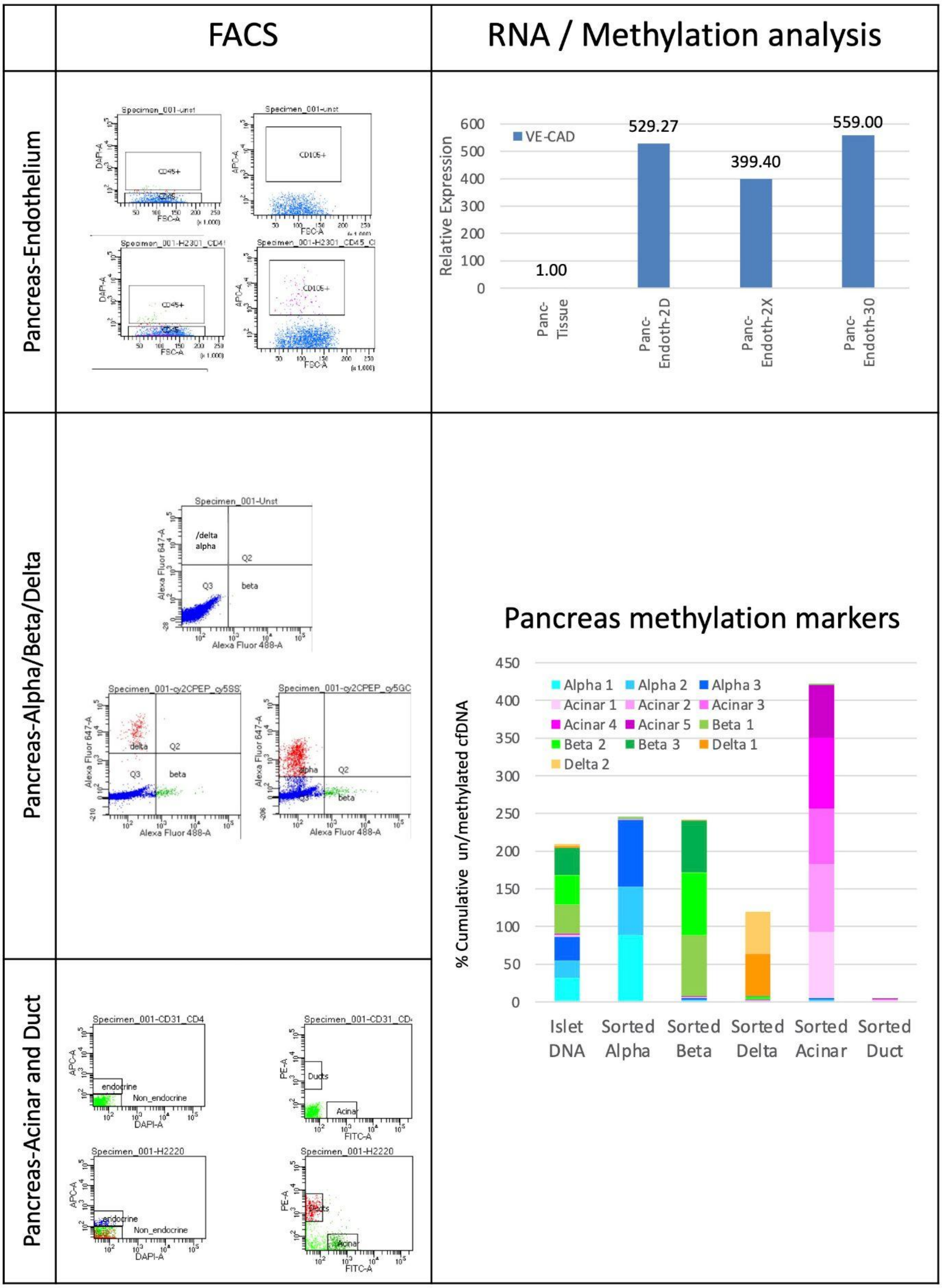
FACS
RNA / Methylation analysis
Pancreas-Endothelium
Pancreas-Alpha/Beta/Delta
Pancreas-Acinar and Duct
VE-CAD
Relative Expression
1.00
529.27
399.40
559.00
Panc-Tissue
Panc-Endoth-2D
Panc-Endoth-2X
Panc-Endoth-30
Pancreas methylation markers
% Cumulative un/methylated cfDNA
Alpha 1
Alpha 2
Alpha 3
Acinar 1
Acinar 2
Acinar 3
Acinar 4
Acinar 5
Beta 1
Beta 2
Beta 3
Delta 1
Delta 2
Islet DNA
Sorted Alpha
Sorted Beta
Sorted Delta
Sorted Acinar
Sorted Duct
Specimen_001-Unst
Alexa Fluor 647-A
Alexa Fluor 488-A
/delta alpha
Q2
Q3
beta
delta
alpha
Specimen_001-H2220
endocrine
Non_endocrine
Ducts
Acinar
DAPI-A
FITC-A
APC-A
PE-A

<table>
<tr><th></th><th>FACS</th><th>RNA / Methylation analysis</th></tr>
<tr><td>Tongue-Epithelium</td><td>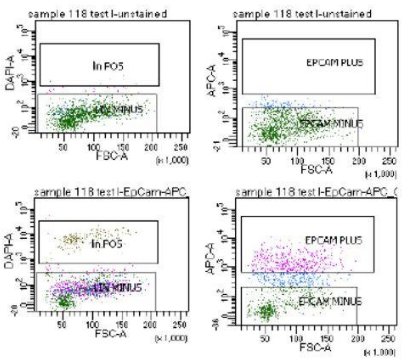
</td><td>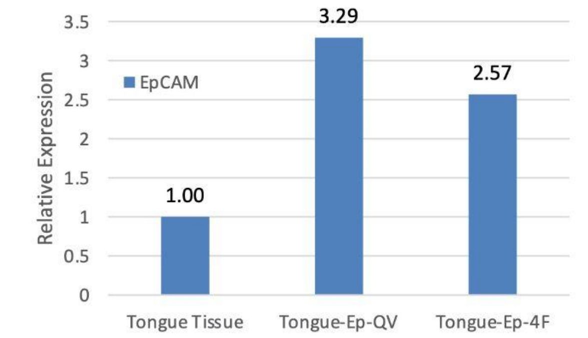
</td></tr>
<tr><td>Tongue_base-Epithelium</td><td>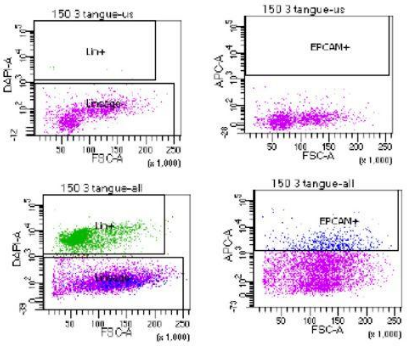
</td><td>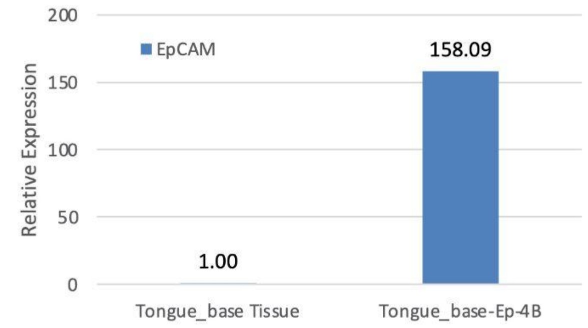
</td></tr>
<tr><td>Larynx Epithelium</td><td>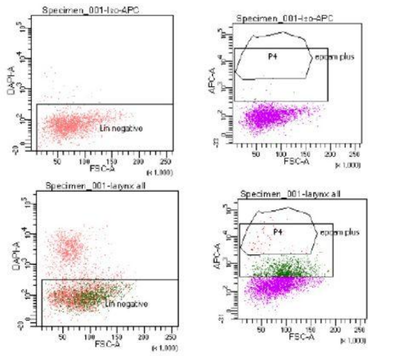
</td><td>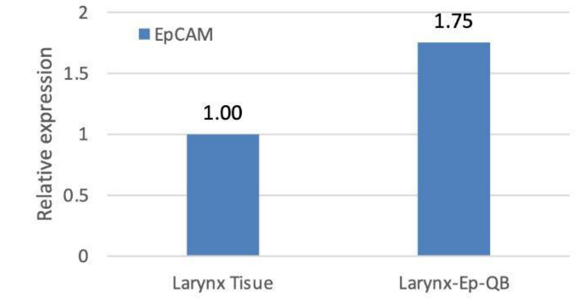
</td></tr>
<tr><td>Esophagus Epithelium</td><td>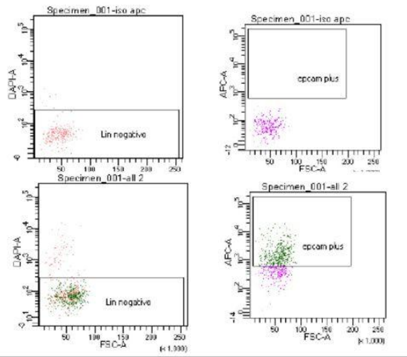
</td><td>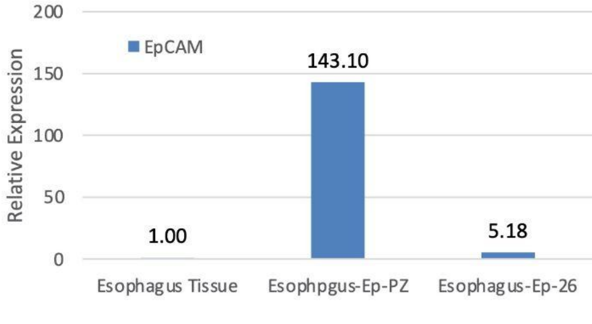
</td></tr>
</table>

<table>
<tr><th></th><th>FACS</th><th>RNA / Methylation analysis</th></tr>
<tr><td>Pharynx Epithelium</td><td>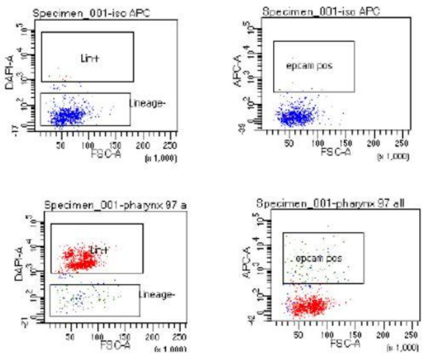</td><td>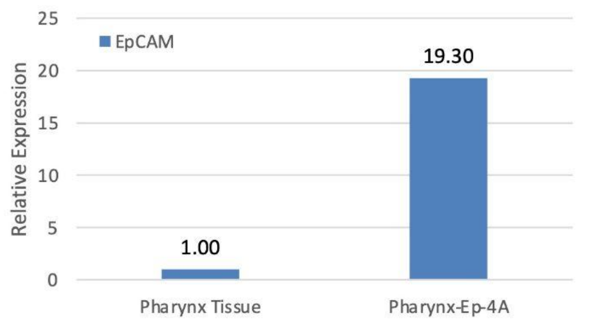
</td></tr>
<tr><td>Lung Bronchus Epithelium</td><td>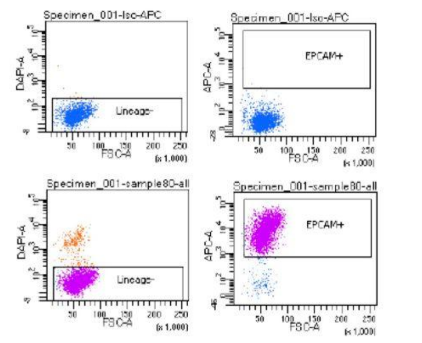</td><td>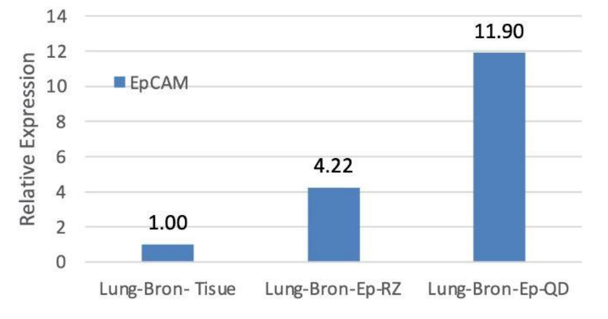
</td></tr>
<tr><td>Lung Alveolar Epithelium</td><td>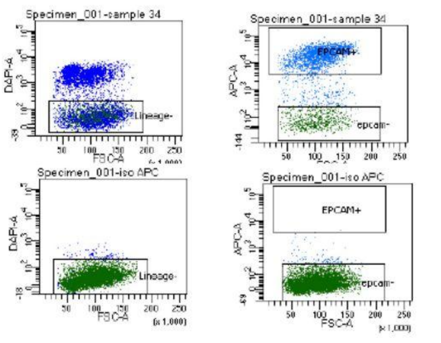</td><td>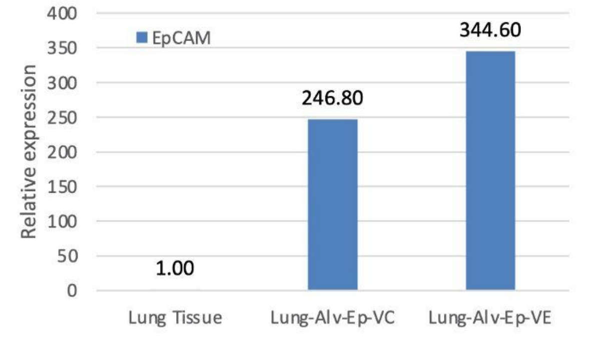
</td></tr>
<tr><td>Prostate Epithelium</td><td>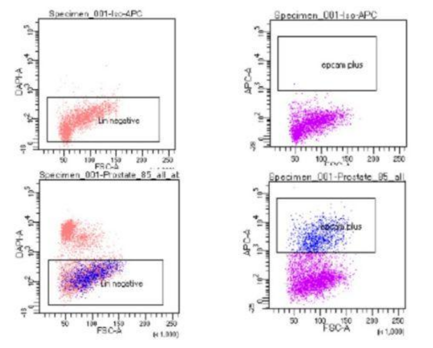</td><td>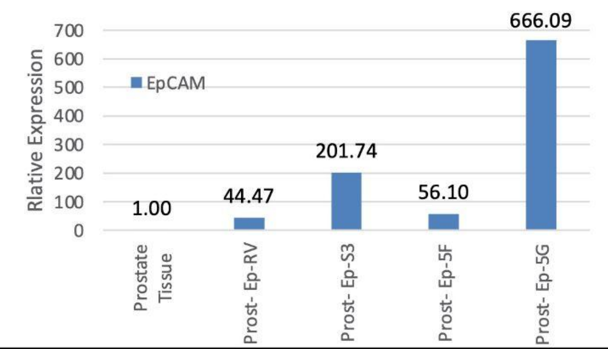
</td></tr>
</table>

	FACS	RNA / Methylation analysis
Bladder-Epithelium	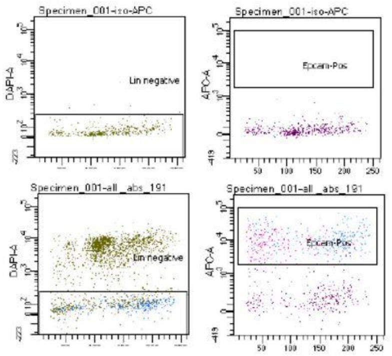	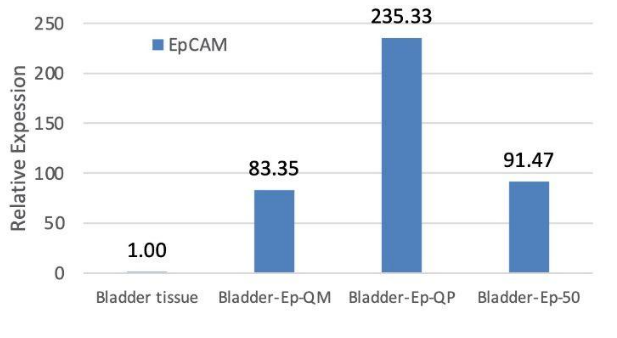
Gallbladder-Epithelium	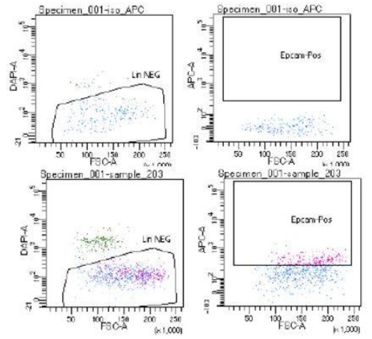	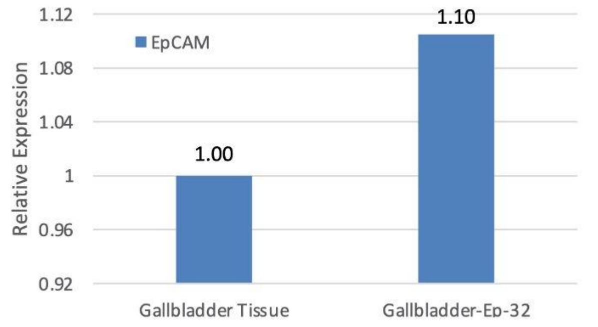
Gastric-Epithelium	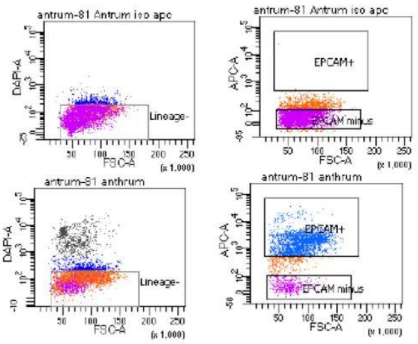	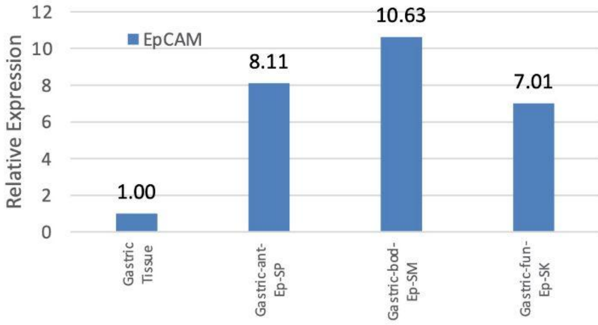
Colon-Epithelium	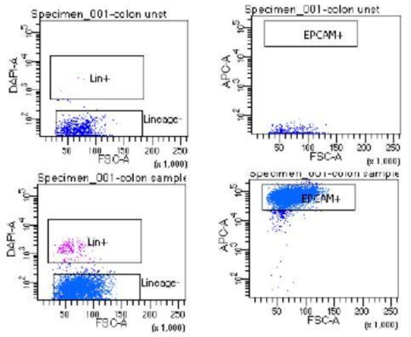	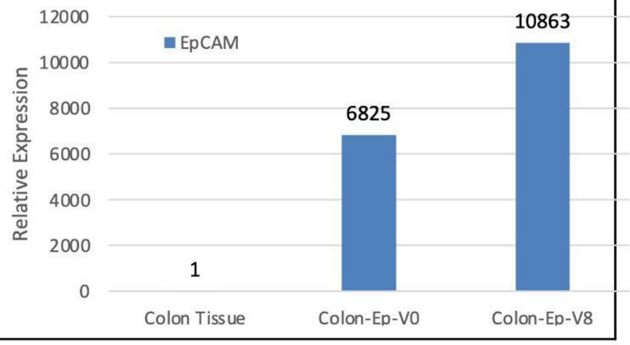

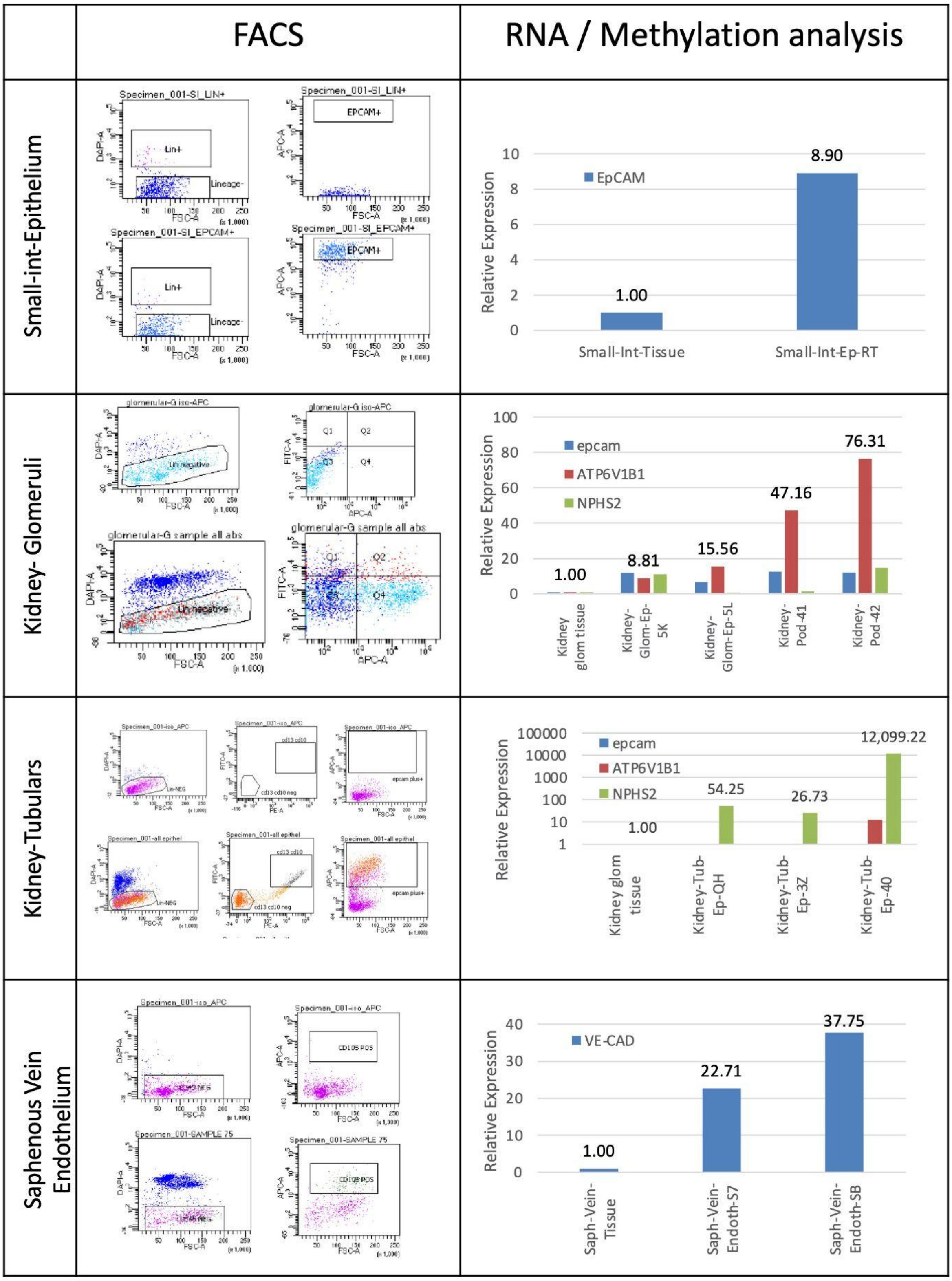
FACS
RNA / Methylation analysis
Small-int-Epithelium
Relative Expression
EpCAM
8.90
1.00
Small-Int-Tissue
Small-Int-Ep-RT
Kidney- Glomeruli
Relative Expression
epcam
ATP6V1B1
NPHS2
76.31
47.16
15.56
8.81
1.00
Kidney glom tissue
Kidney-Glom-Ep-5K
Kidney-Glom-Ep-5L
Kidney-Pod-41
Kidney-Pod-42
Kidney-Tubulars
Relative Expression
epcam
ATP6V1B1
NPHS2
12,099.22
54.25
26.73
1.00
Kidney glom tissue
Kidney-Tub-Ep-QH
Kidney-Tub-Ep-3Z
Kidney-Tub-Ep-40
Saphenous Vein Endothelium
Relative Expression
VE-CAD
37.75
22.71
1.00
Saph-Vein-Tissue
Saph-Vein-Endoth-S7
Saph-Vein-Endoth-SB

<table>
<tr><th></th><th>FACS</th><th>RNA / Methylation analysis</th></tr>
<tr><td>Breast epithelial (luminal and basal)</td><td>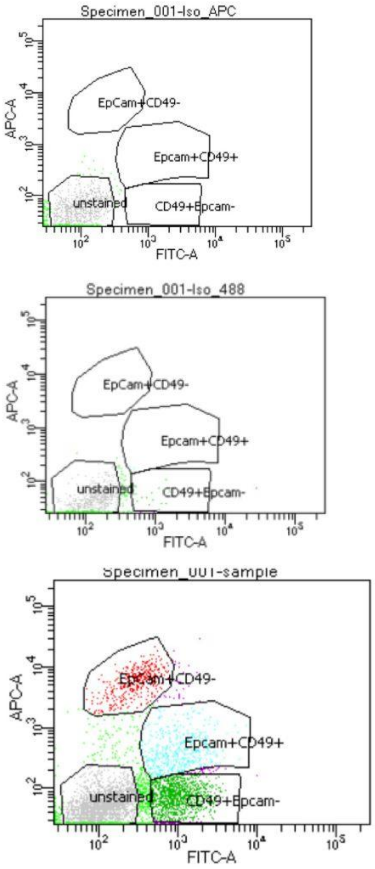
</td><td>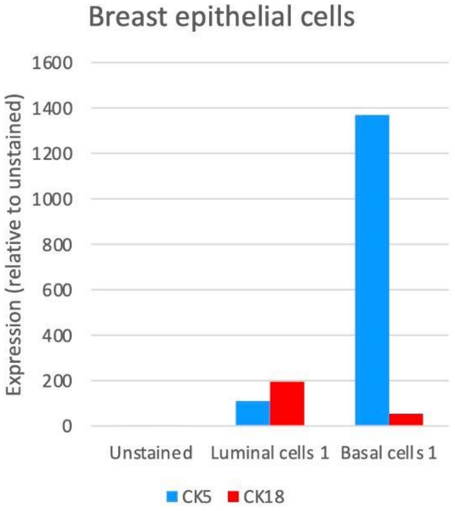
</td></tr>
</table>

	FACS	RNA / Methylation analysis
T and B Cells	see figures 1–2 below	see figure 3 below

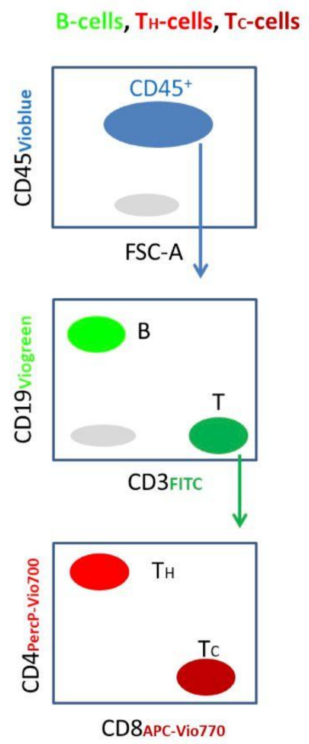

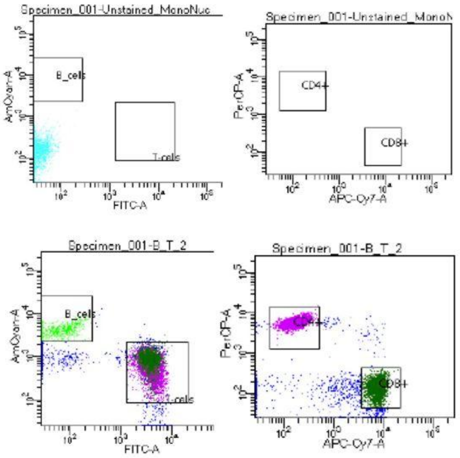

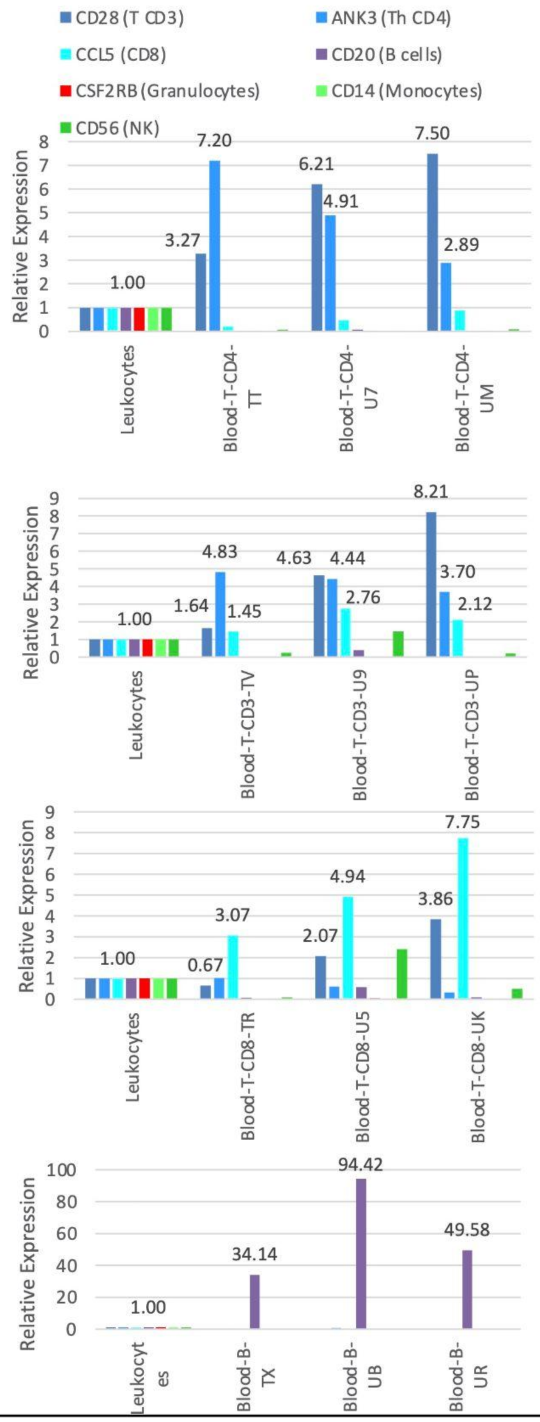

	FACS	RNA / Methylation analysis
Monocytes NK and Granulocytes cells		

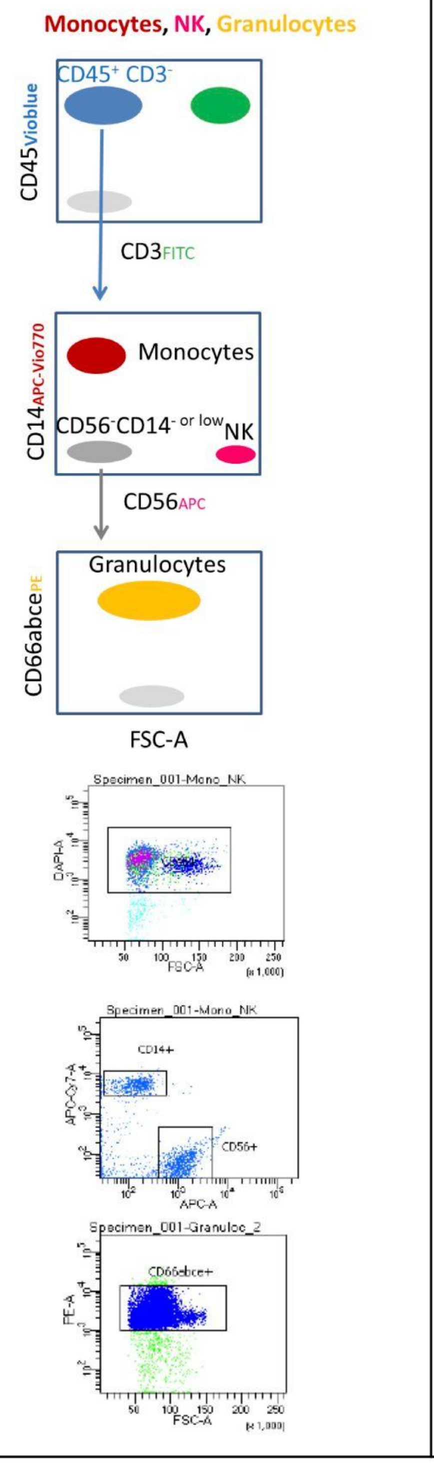

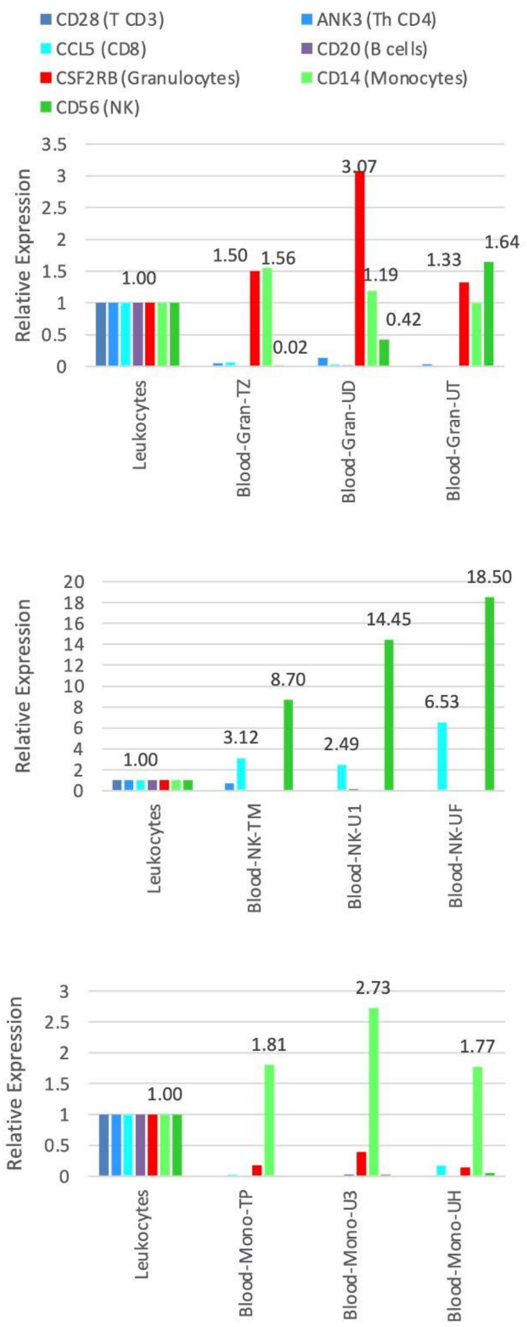

	FACS
Naïve and central memory CD4-T-cells	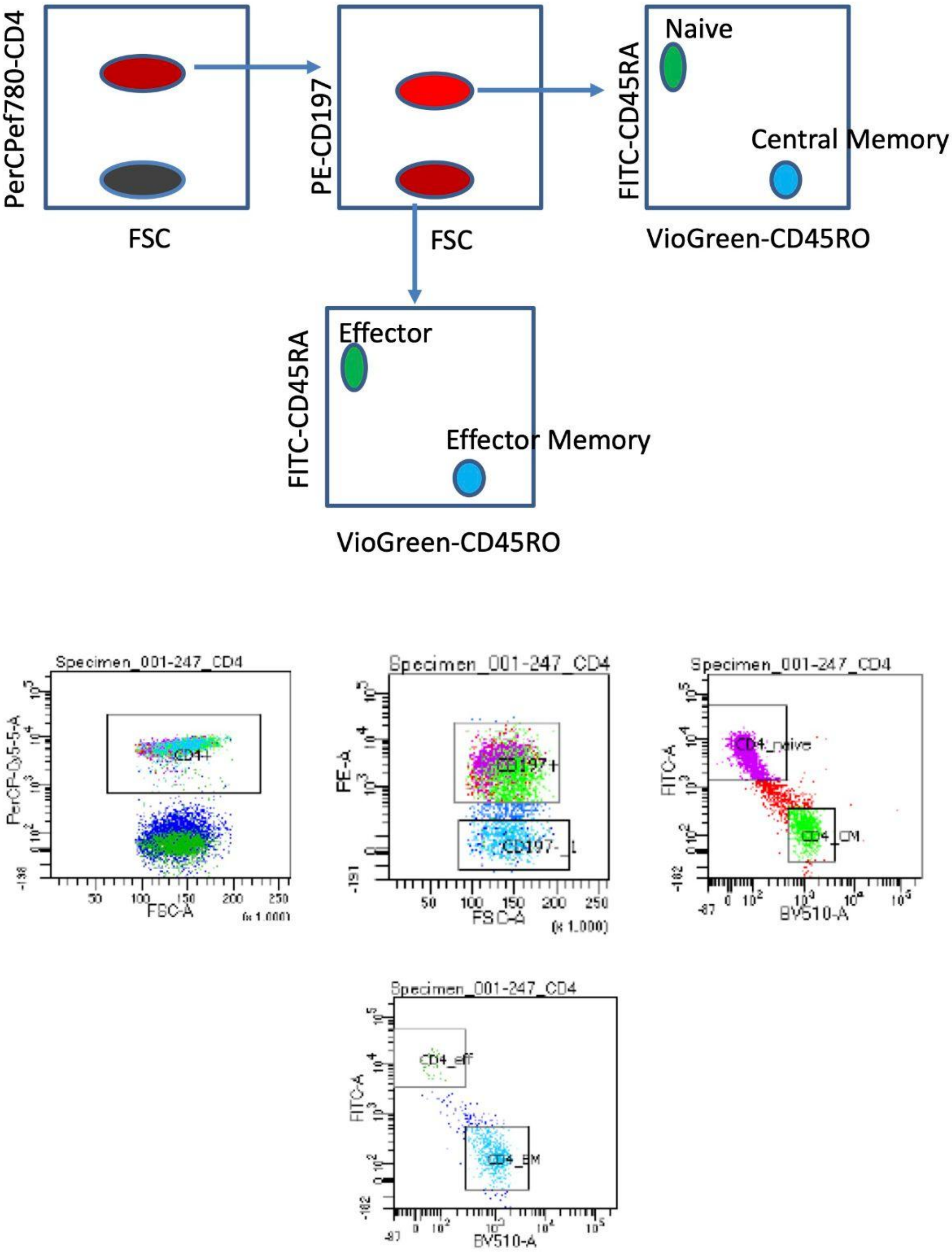

	FACS
Naïve and Effector memory CD8-T-cells	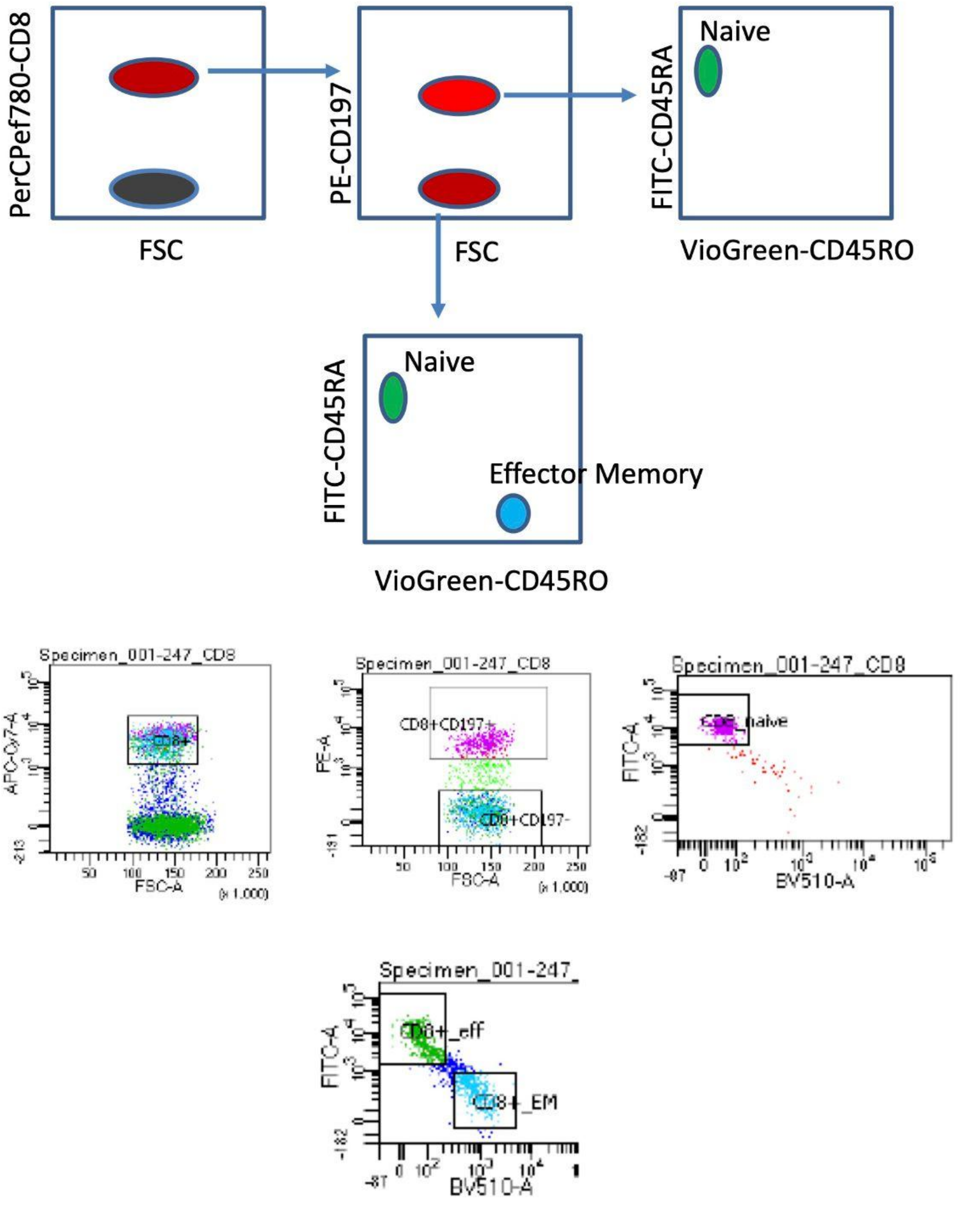

	FACS
memory B-cells	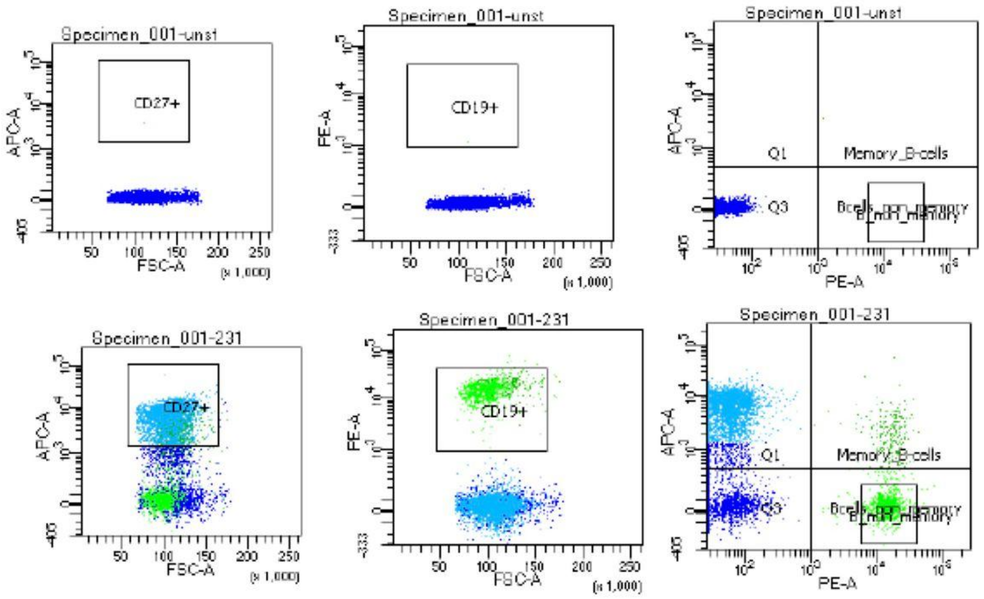

RNA analysis - Lonza cells

Smooth muscle cells	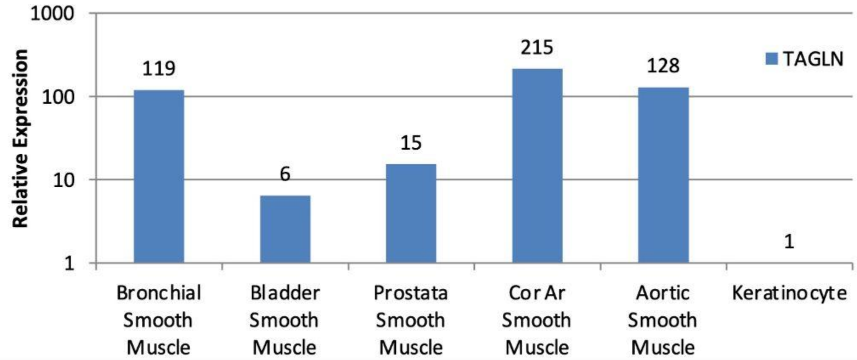
Keratinocytes	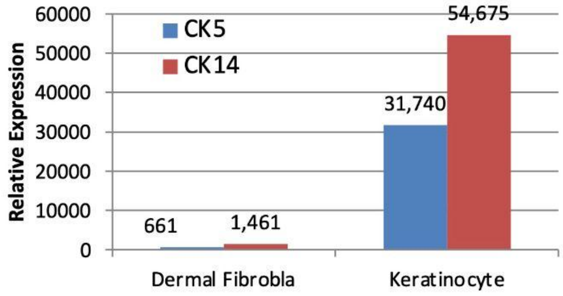
Cardiac Fibroblasts	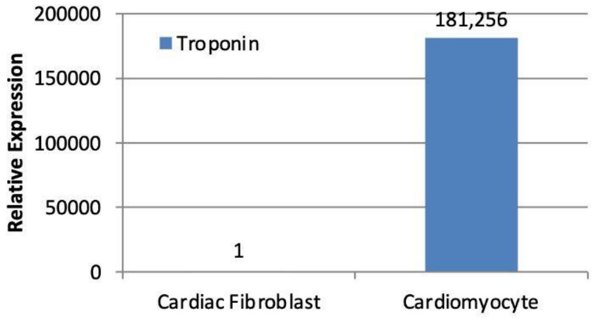
Aortic Endothelium	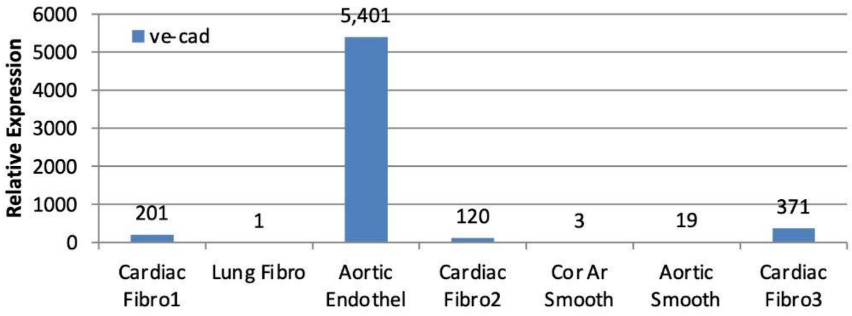

Genome-wide segmentation to methylation blocks

We devised a computational heuristics for selecting the top genomic regions, differentiating between the samples of a target cell type and all other samples, based on their average CpG methylation levels.

The algorithm is a simple Dynamic Programming segmentation algorithm, that breaks the genome into continuous genomic regions (or blocks) showing similar methylation levels across one or more CpGs. Roughly speaking, the algorithm finds a segmentation that minimizes the variance of methylation levels around each segment's mean. For example, a segmentation of the CpGs in a 4,500 bp window (85 CpGs), based on their colon epithelial methylation levels might look like that:

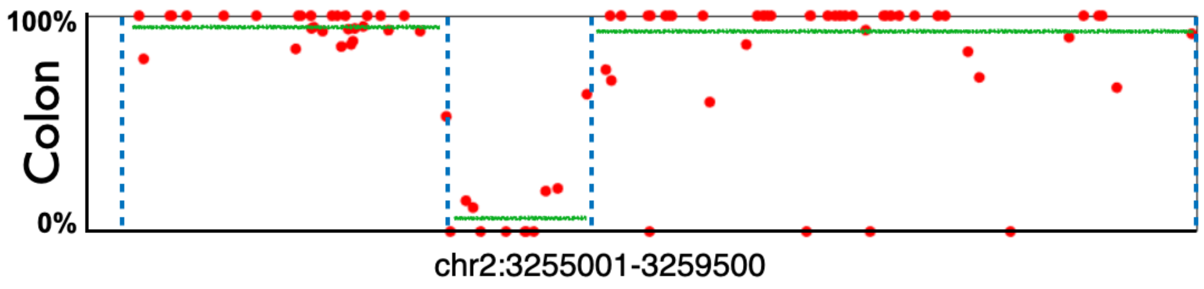

With blue dotted lines marking the segments, and green lines marking the mean methylation values in each segment.

We model CpG methylation using a probabilistic model, whereas all CpGs in the the i'th block of the k'th sample are binary (Bernoulli) random variables with a joint probability θ_i^k of being methylated.

Given a segmentation, this probability θ_i is estimated using the frequency of methylated CpGs in the region, as counted across all overlapping fragments. Formally, the maximum-likelihood estimator (MLE) of θ_i , could be written as:

$$\hat{\theta}_i = \frac{(N_C)_i}{(N_C)_i + (N_T)_i}$$

where $(N_C)_i$ is the number of methylated observations i'th block i, and $(N_T)_i$ is the number of unmethylated observations. This expression is equivalent to the averaging the methylation levels in all CpGs within the block, weighted by the number of observations for each CpG.

In practice, we allow some pseudocounts α_C, α_T for methylated and unmethylated observations, yielding the Bayesian estimator:

$$\hat{\theta}_i = \frac{(N_C)_i + \alpha_C}{(N_C)_i + (N_T)_i + \alpha_C + \alpha_T}$$

We segment the genome using a Dynamic Programming algorithm that maximizes the likelihood of the data (observations). For this, the likelihood is calculated for each block.

Simply put, the likelihood of each observed CpG is θ_i in cases it was methylated, and $1 - \theta_i$ otherwise. As there are $(N_C)_i$ methylated observations, and $(N_T)_i$ unmethylated ones, the likelihood can be written as:

$$l_i = (\hat{\theta}_i)^{(N_C)_i} \cdot (1 - \hat{\theta}_i)^{(N_T)_i}$$

or in log-scale

$$ll_i = \Sigma \ \ ((N_C)_i \ \cdot \log(\hat{\theta}_i) + (N_T)_i \ \cdot \log(1 - \hat{\theta}_i))$$

In most cases, we will segment multiple samples together, using shared segments across all channels. The colon sample from above might be segmented differently when heart and blood B cells are combined as additional channels:

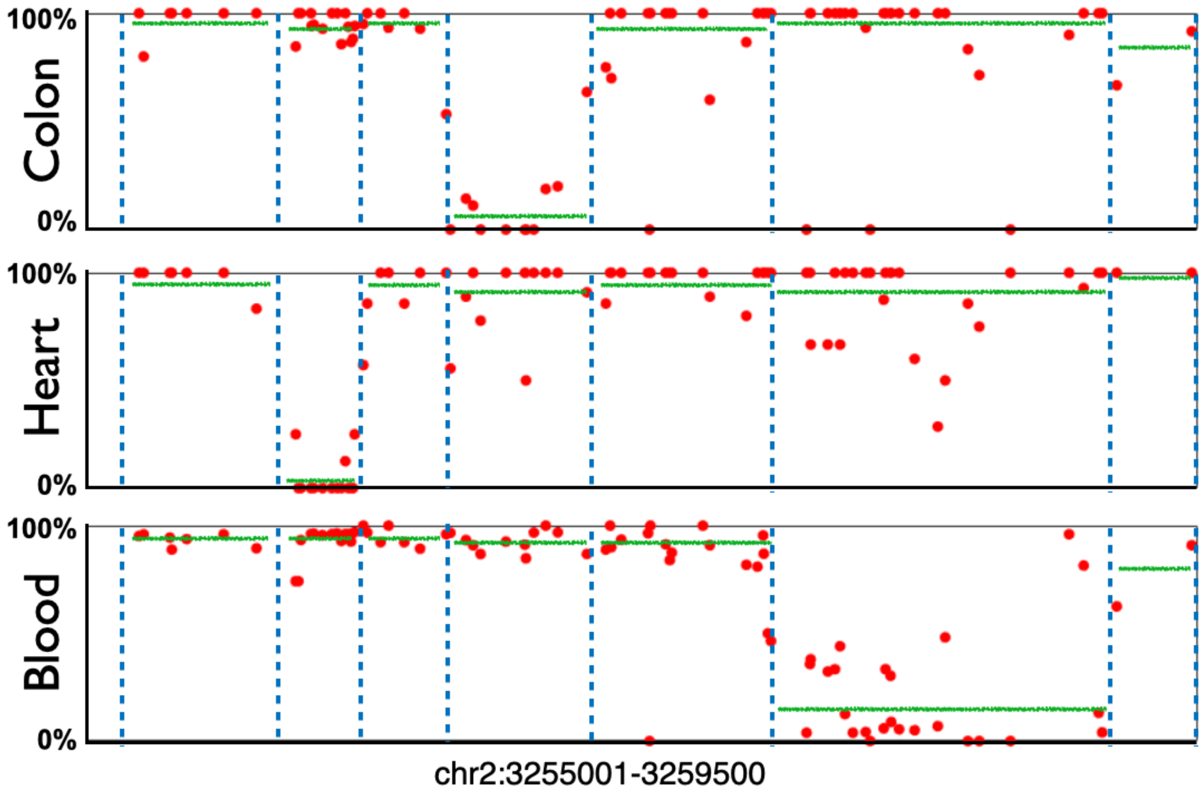

Yet, the mathematical formulations are identical, adding up the log-likelihood values at the different samples 1…K:

$$score(block_i) = ll_i = \Sigma^K_{k=1}((N_C)_i^k \cdot \log(\hat{\theta}_i^k) + (N_T)_i^k \cdot \log(1 - \hat{\theta}_i^k))$$

Finally, we are ready to review the Dynamic Programming segmentation algorithm maximizing these scores. The algorithm works by solving each problem using pre-calculated solutions for smaller, easier problems. Here, we break down the segmentation of a full chromosome to smaller problems of segmenting prefixes. For this we maintain a $1 \times N$ table T, holding the scores of the optimal segmentation for each prefix. For example $T[1]$ holds the (trivial) segmentation of the first CpG into one single block. $T[2]$ holds the score of the

optimal segmentation of the two first CpGs, which can either be grouped as one segment, or broken into two separate segments. While the latter score should be calculated from scratch, the score of two separate segments could be calculated using the predefined $T[1]$:

$$T[2] = \max\{score(block[1,2]),\ T[1] + score(block[2,2])\}$$

Similarly, $T[3]$ could be calculated using $T[1]$ and $T[2]$:

$$T[3] = \max\{score(block[1,3]),\ T[1] + score(block[2,3]),\ T[3] + score(block[3,3])\}$$

Namely, we compute the score of the full segment [1,3]; or the optimal segmentation up to position 1, plus the score of [2,3]; or the optimal segmentation up to position 2, plus the score of [3,3]; and take the maximum.

Generally speaking, the table is updated for each i from 1 to N, as:

$$T[i] = \max_{i'<i}\{T[i'] + score(block[i' + 1,\ldots,i])\}$$

Namely, the optional segmentation of [1,i] could be found by considering the optimal segmentation of [1,i-1] plus the block [i,i]; or the optimal segmentation of [1,i-2] plus the block [i-1,i]; or the optimal segmentation of [1,i-3], plus the block [i-2,i], etc. In practice, we assume some maximal segment length (e.g. 5000 bp) for efficiency reasons.

Eventually, $T[n]$ will hold the score of the **optimal segmentation of the entire genome**.

This algorithm uses an independence assumption, by which the score of a segment [i,j] is independent of the segmentation of the previous positions [1,i-1]. This allows the algorithm to calculate each segment just once, and find the optimal segmentation score of each prefix based on its predecessors.

Importantly, we also maintain a "traceback" table, which records for each i how the optimal score for the segmentation of [1,i] was obtained, or more specifically, at which position the last block started. When $T[n]$ is completed, we can use this traceback table to retrieve the optimal segmentation from the end backwards.

Overall the algorithm is not influenced by the initialization or the relative position within each chromosome and is guaranteed to find the optimal segmentation genome-wide, given the score and independence assumptions described above.

The human genome was segmented into 7,264,350 continuous homogeneous blocks. The histograms show the number of segmented blocks as a function of the number of CpGs they contain (left), or as a function of their length in bases (right). In addition to the 2,746,623 blocks of length 3-30 CpGs (plotted here), there were additional 3,271,607 singleton blocks (composed of one CpG), and 1,185,719 blocks of two CpGs, as well 60,401 of >30 CpGs.

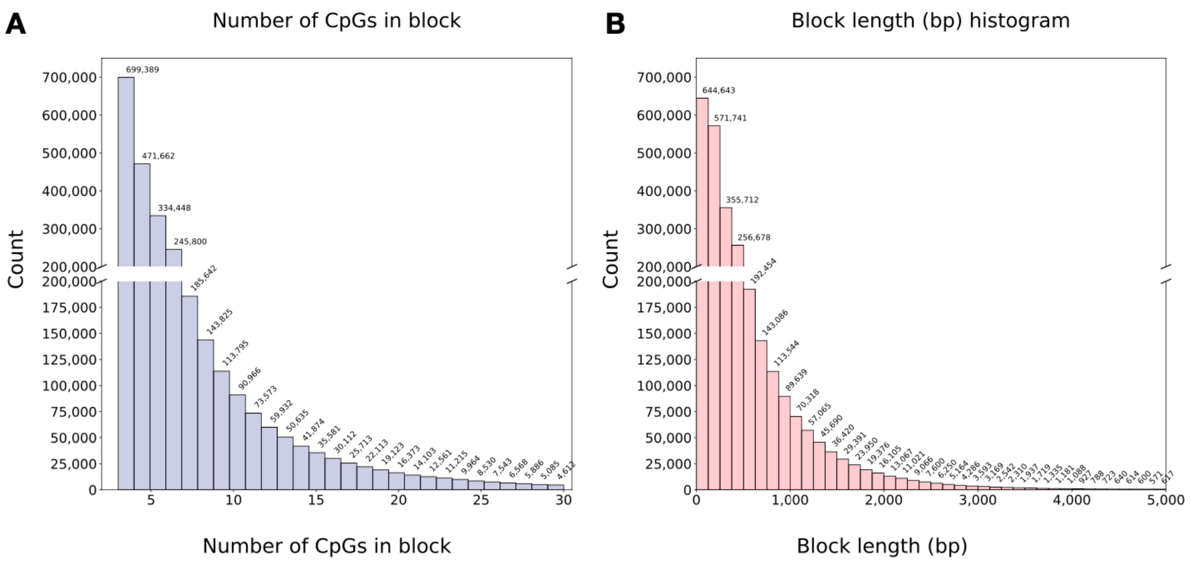

Selection of type-specific markers

We devised a computational heuristics for selecting the top genomic regions, differentiating between the samples of a target cell type and all other samples, based on their average CpG methylation levels.

For this, we first identify genomic regions (segmented blocks) that cover ≥5 CpGs, with length varying between 10 to 500bp. We then calculated for each such genomic region the average methylation across all CpGs in all fragments, for each sample.

Our goal is to identify regions that show the greatest differences in DNA methylation patterns in the target group of samples, compared to all other background samples. This could be obtained by computing the difference in average methylation, or “delta beta”, between the target and background samples. Yet, such an approach is not ideal for deconvolution purposes, because few other samples in the background group might have similar methylation patterns, but they will have a limited effect on the overall average of the >200 samples in the background group, due to their small number.

Instead, it is reasonable to adopt a “maximal margin” approach, where the maximal difference is sought between all target samples vs. all background samples. In the case of U-type markers, which are unmethylated in the target group, this translates to comaprint the most methylated sample in the target group, vs. the least methylated sample in the background group. As all max/min scores, this approach is sensitive to outliers, and so we sought a more flexible “soft margin” margin approach. Specifically, we compute the methylation value at the 75th percentile in the sample group, which is the most methylated sample for types of 3 samples or less (as before), but only the 2nd methylated for types of four through seven samples, thus allowing one outlier. Similarly, we compute the 2.5th percentile in the background samples, thus allowing a small number of ~5 outliers in that group (marked by dotted blue line). In the plot below, the min/max threshold are marked by

solid lines, whereas the 75th percentile of the target samples are marked by a dotted red line, and the 2.5th percentile of the background group by a dotted blue line.

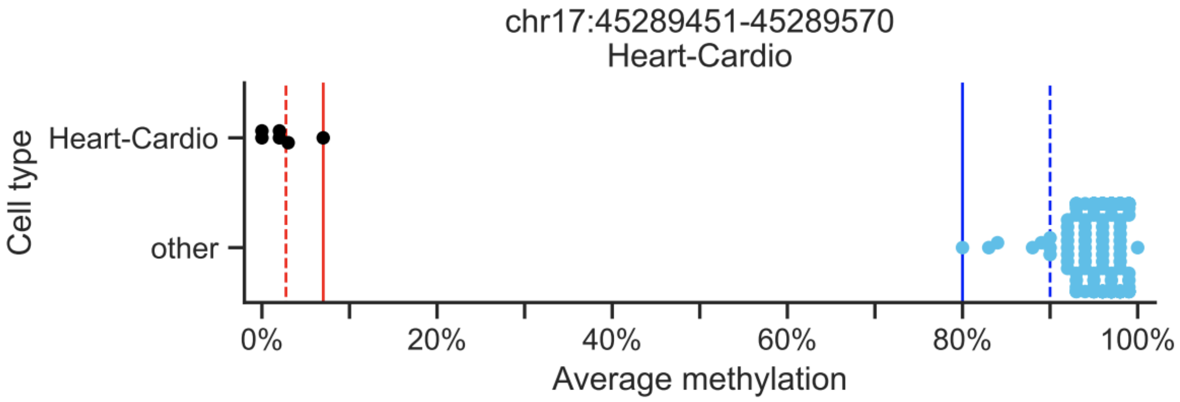

In genomic regions where the read coverage is very low, the average methylation level is based on few observations and the estimation error is relatively high. For example, assuming a simple independence model, where each observation (one CpG in one sequenced fragment) is a Bernoulli random variable sampled from a probability parameter *p*, the std of the sample mean is sqrt(p*q/n), with *q=1-p*, and *n* the number of observations. Assuming *p* in the range [5%,15%], a value of n=25 suggests an estimation error of ~10%, which is sufficient for the marker selection process. We therefore set the minimal number of observations, per block, to a default value of n=25. In cases where this threshold is not achieved, and the average methylation might be poorly estimated, we set a default threshold of 0.5, which is neither methylated nor unmethylated, and thus actively narrows the margin between the target and background groups. This allows the algorithm to select such markers only when the number of low-coverage regions is limited, and as a last resort. Plotted below, is a colon epithelial cells marker (chr1:167870100-167870517), in which one cardiomyocyte sample contains only 23 observations (all methylated) and so an imputed value of 0.5 is set.

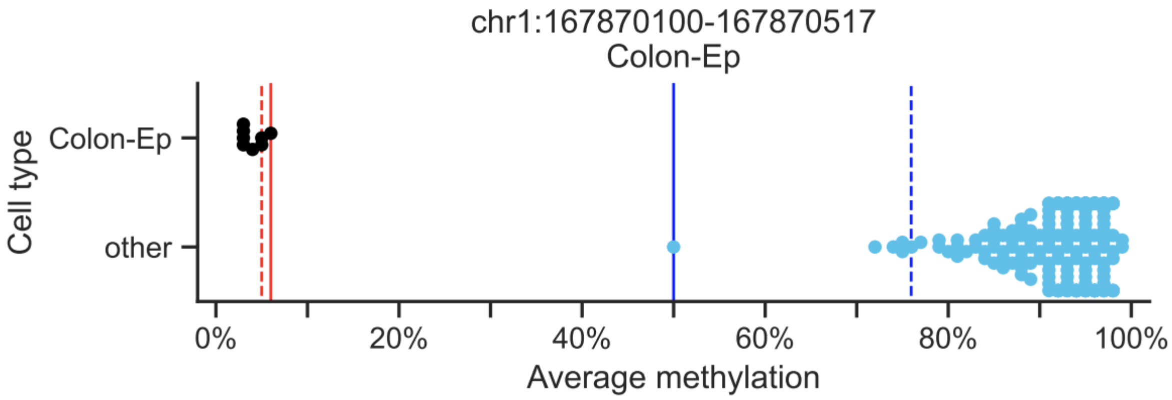

UXM fragment-level deconvolution algorithm

UXM is a fragment-level deconvolution algorithm for next-generation sequencing data following enzymatic or bisulfite conversion.

The algorithm is based on fragment-level classification of fragments, according to their relative proportion of methylated and unmethylated CpGs. Specifically, we calculate the proportion of “mostly unmethylated” (U), “mostly methylated” (M), and mixed (X) fragments at each genomic region. For this, the algorithm considers all fragments with k=4 CpGs or more, and calculates the percent of methylated CpGs per read.

Given a UXM threshold of 25%,75%, fragments with average methylation ≤ 25% are classified as “U”, fragments > 25% but < 75% as “X”, and fragments with ≥ 75% methylated CpGs as “M”. Fragments exceeding the marker boundaries to the left/right are trimmed and only the CpGs within the marker region are considered. Similarly, missing values are not counted towards the k=4 CpGs threshold.

This can be visualized using wgbstools, with the command “wgbstools vis -r chr2:3255400-3256300 Heart-Cardiomyocyte-Z0000044N.pat.gz --min_len 4 --text --uxm 0.75 --strict --strip”

```
chr2:3255400-3256300 - 901bp, 23CpGs: 2333059-2333082
intron  TSSC1
Heart-Cardiomyocyte-Z0000044N
Methylation average: 17%
UXM 75/25 [14/2/3] [73.7%/10.5%/15.8%]
+++++++++++++++++++++++
  UUUU   UUUUUU  MMMMM
   UUUUUU UUUUUUUU
   UUUUUU  UUUUUUUUU
   UUUUUUU    XXXXXX
   UUUUUUU        MMMM
    UUUUUUUU      MMMMM
     UUUUUUUUUUUUU
      XXXXXXXXXXXXXXXX
      UUUUUUUUU
      UUUUUUUUUUUUUUU
         UUUUUUUUU
         UUUUUUUUU
```

Compared to the original data (without UXM classification): “wgbstools vis -r chr2:3255400-3256300 Heart-Cardiomyocyte-Z0000044N.pat.gz --min_len 4 --text --strict --strip”

```
chr2:3255400-3256300 - 901bp, 23CpGs: 2333059-2333082
intron  TSSC1
Heart-Cardiomyocyte-Z0000044N
Methylation average: 17%
+++++++++++++++++++++++
  CTTT   TTTTTT  CCCCC
   CTTTTT TTTTTTTT
   TTTTTT  TTTTTTCTT
   TTTTTTT    TTTTCC
   TTTTTTT        CCCC
    TTTTTTTT      CCCCC
     TTTTTTTTTTTTT
      TTTTTT......TCCC
      TTTTTTTCT
      TTTTTTTT...TTCC
         TTTTTTTTT
         TTTTTTTTT
```

As the first row demonstrates, the CTTT fragment (25% meth) is classified as U, whereas the third one (CCCCC) as M. The two X reads are TTTTCC with 2 methylated CpGs out of 6 (33%), and TTTTTT......TCCC with 3 methylated CpGs out of 10 (30%; not including 6 CpGs, that are not covered by the paired-end 150bp-long reads, and are marked by “.” here). Overall, this sample (Heart-Cardiomyocyte-Z0000044N) contains 14 U fragments, 2 X fragments, and 3 M fragments at this genome region (chr2:3255400-3256300, part of the region shown in Figure 1).

UXM performs this calculation across an input set of marker regions, for every cell type. For example, the proportions of the “U” reads at the Cardiomyocyte marker chr17:45289451-45289570 (Figure 3C) are shown below:

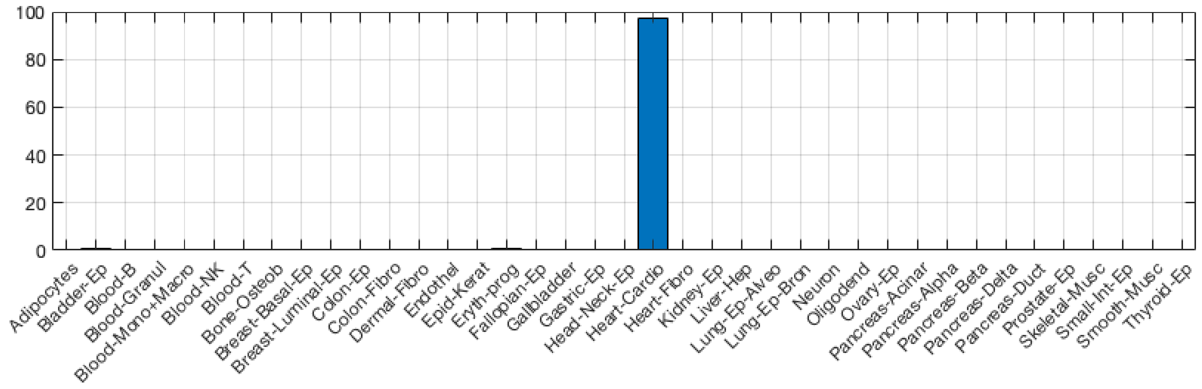

with 97.5% (or 153 out of 157) fragments with ≥4 CpGs in heart cardiomyocyte samples are classified as U, compared to less than 1% in other cell types.

Obviously, these parameters, of the minimal number of CpGs in analyzed fragments, or the exact threshold used for U/X/M classification, could be changed to maximize the differences between fragment-level differences in methylation across different cell types.

Overall, we constructed a reference atlas *A*, where the $A_{i,j}$ cell holds the U proportion of the i'th marker across fragments from the j'th cell type. Our atlas typically contained 1,232 markers, which include 953 cell type-specific markers, as well as additional 279 markers for cell type combinations (e.g. breast basal and luminal epithelial cells). These are reported in Table S4, and visualized in the following heatmap. The reference atlas can be altered. For example, in tissue-specific deconvolutions, it is advised to only include markers of the relevant cell types, to minimize noise. Similarly, UXM allows deconvolution of additional cell types. In plasma related analyses for example, we considered an additional set of 25 megakaryocytes markers.

Notably, the atlas can also include hyper-methylated markers that are unmethylated in all cell types but methylated in one (or few) cell types. Here, one should include the proportion M fragments in each cell type.

We also tried including all U/X/M types for each marker, to a total of 1,232x3 rows in *A*, or only include two of the three types per marker (as the proportions sum up to 1 per marker, so are linearly dependent). These extended markers did not improve the deconvolution accuracy, possibly due to additional noise from the "non-relevant" types that were included.

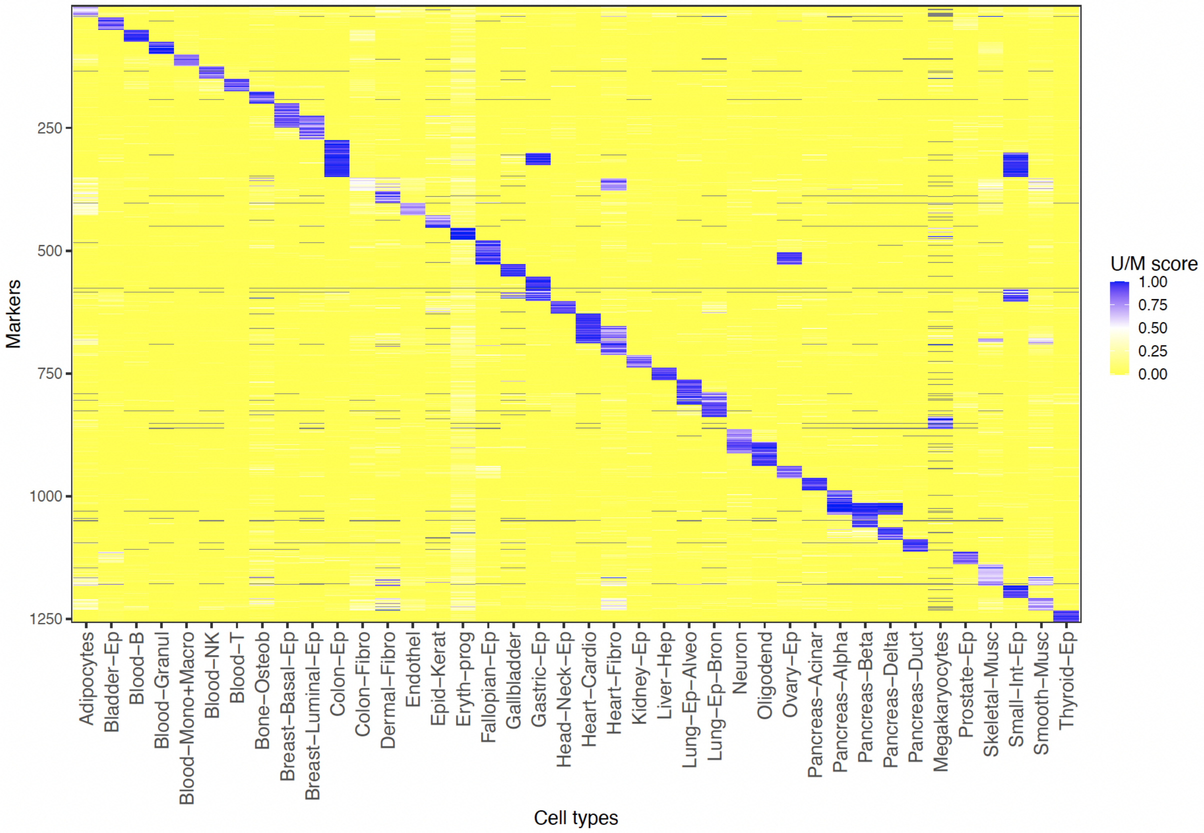

For deconvolution, UXM then calculates the proportion of U/X/M fragments in an input sample of cell-free DNA methylation, obtained from plasma, or a whole-tissue sample, and constructs a vector *b* dimensions 1,232x1. Finally, we use NNLS deconvolution algorithm, similar to the one we used for deconvolution with the DNA methylation atlas by Moss et al, 2018, for 450K/EPIC-array data (available here: https://github.com/nloyfer/meth_atlas). Mathematically, this is a constrained multiple linear regression model that aims to approximate the input vector *b* as a non-negative combination of the atlas cell types. Formally, we seek the coefficient vector *x* that minimizes (using L2 norm) then term $|A \cdot x - b|_2$ subject to non-negativity constraints over *x*, and $\sum_j x_j = 1$.

The mathematical model of this deconvolution problem is based on minimizing the root mean squared residual error across multiple equations (or markers). Yet, one might reason that not all equations should be treated equally, as genomic regions with higher coverage are more reliable and should contribute more to the linear regression score. We therefore included two additional weighting features by which each row (marker) is normalized by the total number (or alternatively, the square root) of fragments in the input sample. Mathematically, this can be formalized by computing the coverage C_i of the input sample *b* in each marker *i*, and minimizing $|diag(C) \cdot A \cdot x - diag(C) \cdot b|_2$. Intriguingly, $diag(C) \cdot b$ is exactly the proportion of U fragments in each region, multiplied by the number of fragments, resulting in the number of U fragments in the input sample in each marker region. While this is not the default parameter of UXM, this weighting was shown to better estimate the

proportions in the coefficient vector *x*, e.g. in leave-one-out runs where an atlas sample is analyzed for purity estimations.

Purity estimations: proportion of unmethylated fragments in various samples

We can assess the sensitivity of each marker by comparing the proportion of unmethylated fragments in samples from the target cell type, vs. samples from other cell types. This can be done using targeted PCR sequencing, for some markers, as shown in Extended Figure S2C, for five amplicons designed for differentially methylated regions in pancreatic cell types and possible contaminants, or be applied to the atlas samples, whereas sequenced fragments with ≥3 or ≥4 CpGs are considered.

This shift to fragment-level analysis allows to suppress biological stochasticity often affecting the methylation state of single CpGs, thus improving the marker specificity, exponentially with the number of sequenced CpGs per fragment.

As shown in Extended Figure S3, by focusing on a single region for each cell type (selected from its set of top 25 markers), the percent of unmethylated fragments in samples from the target cell type is typically above 90%, compared to near 0% proportion in other samples from the same tissue or in its surrounding. Here, we used U/X/M thresholds of [0%,15%] for defining U fragments, (15%,85%) for X fragments, and [85%,100%] for defining M fragments, referring to the percent of methylated CpGs in each sequenced fragment, with a minimal of 4 CpGs per fragment.

It should be noted that these measurements are an underestimate of the sample purity, as different markers show different homogeneity in their methylation patterns across cells. These could be possibly affected by the linear proximity of measured CpGs, their sequence context, cellular location in the tissue (e.g., zonation) or even age of the donor, etc. Accordingly, an unbiased estimation of sample purity can only be obtained from a (theoretical) ideal marker, where 100% of the fragments are unmethylated in the target cell type, compared to 0% in all other samples.

Extended Figure S6 shows these measures for each of the 953 cell type-specific markers. These are organized by cell type (rows) and sorted by delta beta values. For example, the first cardiomyocyte plot shows a near 100% proportion of unmethylated fragments in the 6 cardiomyocyte samples (blue points, with bar showing their average), compared to near 0% of unmethylated fragments in all other 201 samples.

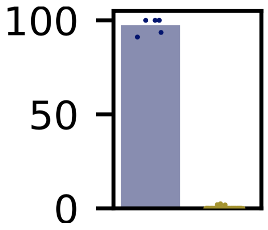

This approach can be extended to any (differentially methylated) genomic regions. For example, we tested by purity of pancreatic cell types by examining cell type-specific genes from a pan-tissue DNA methylation for decomposition of human tissues (EpiSCORE, Zhu et al, Nature Methods, 2022), which were selected using pancreatic single-cell RNA-seq data. We focused on four EpiSCORE genes, including two acinar genes (CTRB2, CELA3B), and two endocrine genes (TTR, FXYD2). For each, we considered methylation levels (purple bars) across all 207 of the atlas samples. We focused on regions near the transcription start site, and selected differentially methylated regions, composed of multiple neighboring CpGs.

We then computed the average methylation levels (orange bars) in various samples from the tissue. We compare these with fragment-level measures, defined as the percent of unmethylated fragments in these regions (green bars).

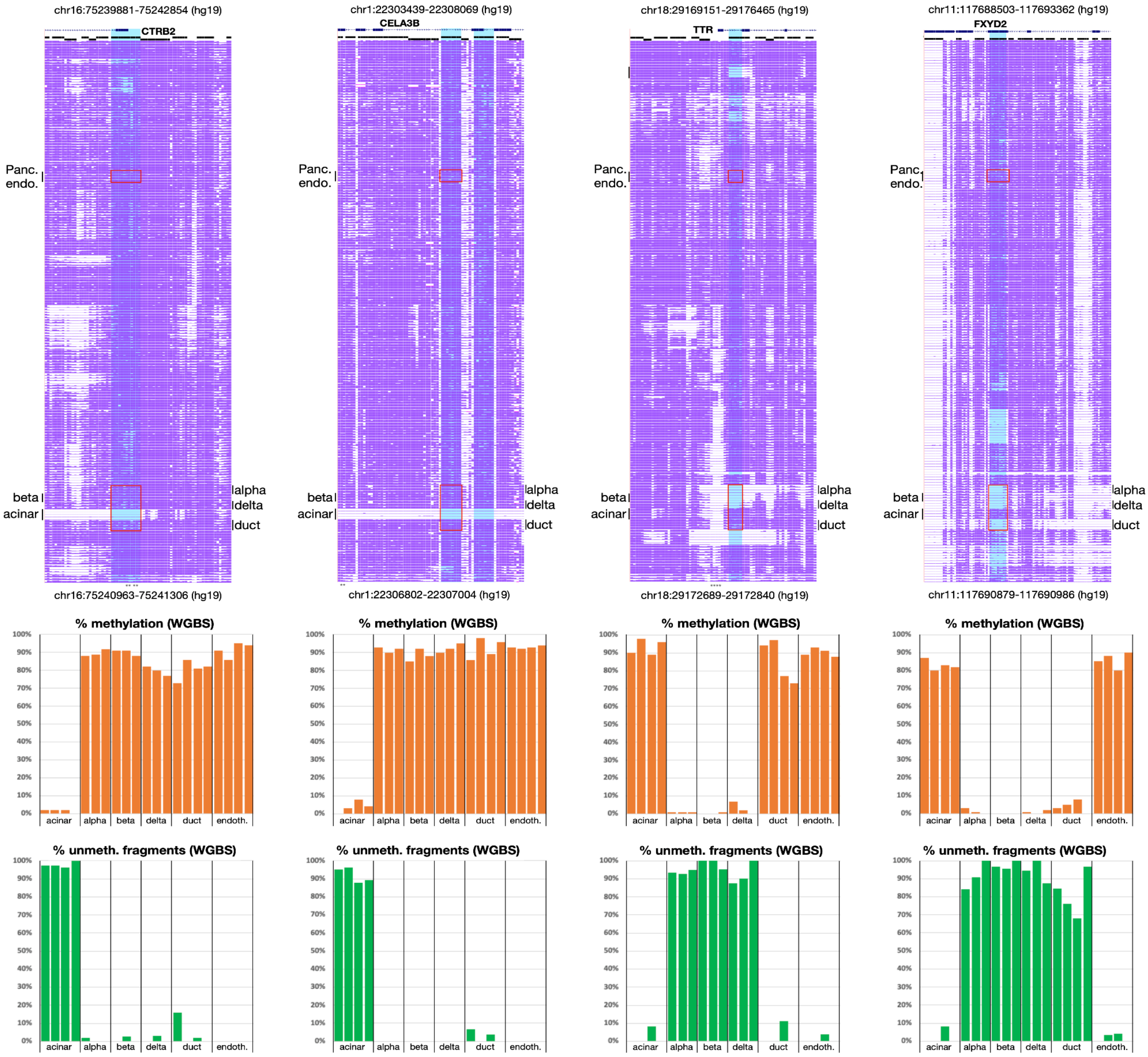

As can been seen for the acinar marker gene CTRB2 (left most), the promoter genomic region at chr16:75240963-75241306 (hg19), is unmethylated in acinar samples, with an average methylation of ~80% in other cell types (orange). A fragment-level analysis shows that ~95% of acinar cell molecules are unmethylated, compared to nearly 0% in other samples (except to one duct sample). Another acinar marker gene CELA3B shows similar trends at an intronic region (chr1:22306802-22307004) near the TSS. Analysis of TTR, a pancreatic endocrine marker identifies a region (chr18:29172689-29172840) exclusively unmethylated in alpha, beta, and delta cells, but also in oligodendrocytes and hepatocytes. Again, fragment-level analysis suggests that ~90% or more of the molecules in endocrine cells are unmethylated. Finally, EpiSCORE uses FXYD2 as a joint marker gene for endocrine and duct cells, in agreement with fragment-level analysis at chr11:117690879-117690986. In all cases, the percentage of unmethylated fragments in other cell types is nearly 0%.

Analysis of cfDNA composition in COVID-19 patients

As shown in Figure 6D, we analyzed published data of COVID-19 patients, from Cheng et al. (Med, 2021, PMID 33521749). We used WGBS atlas with the UXM algorithm described above to deconvolve shallow whole-genome bisulfite sequencing data from 52 hospitalized COVID-19 patients. Consistently with Cheng et al, we identified excessive cell-free DNA fragments from granulocytes, erythrocyte progenitors, lung and liver. Overall, our predictions across all patients are well correlated with the original ones, with a Pearson correlation coefficient of 0.91 (p<7e-243), and are plotted below.

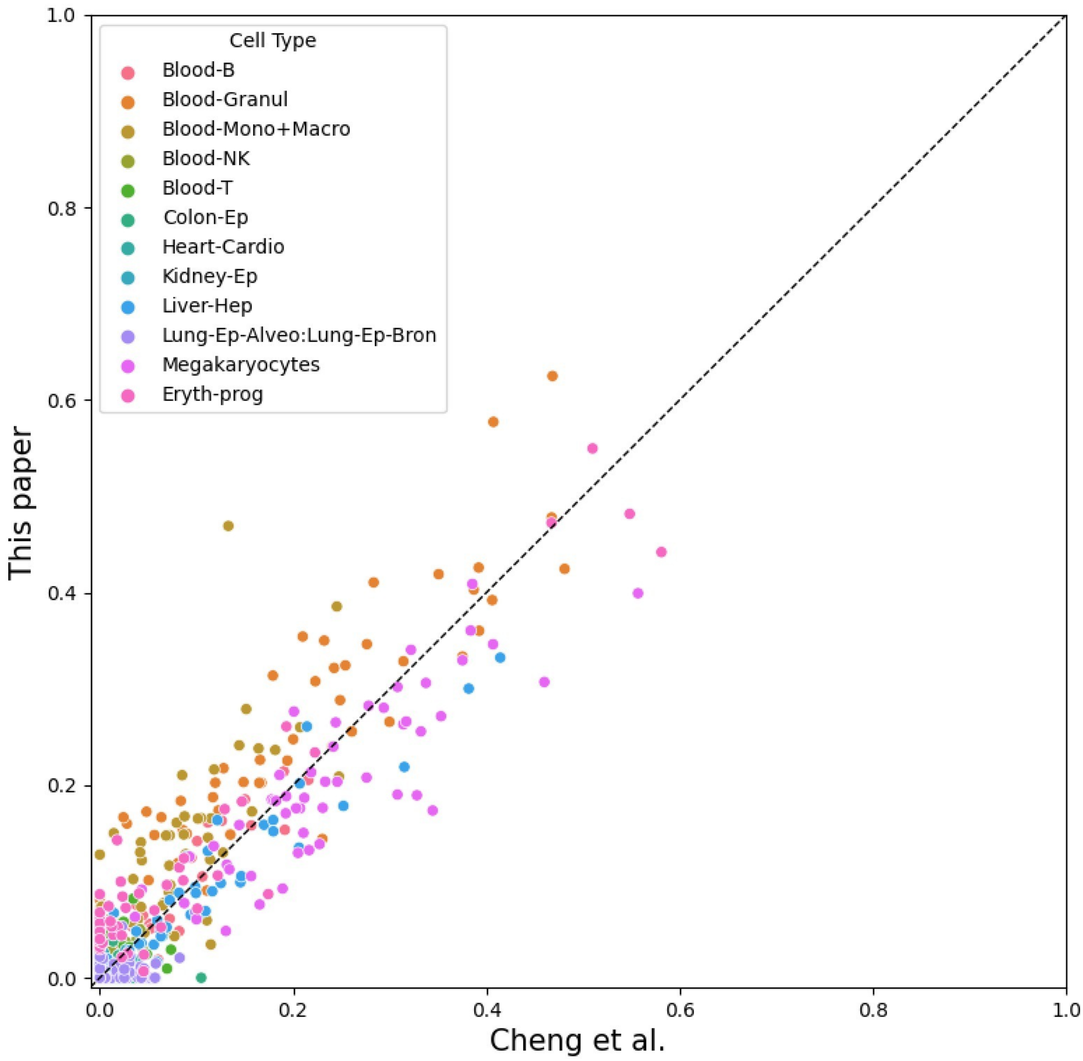

Noneless, we identified a significant contribution of vascular endothelial cells to the cfDNA of these patients, which could not be discovered in the published analysis in the absence of endothelial cell methylome reference. These are presented in Figure 6D.

Dissociation Protocols

Adipocytes

Adipocytes (n = 3) were isolated from fat tissue according to the Collagenase procedure of Rodbell (J. Biol. Chem. 239: 375). In brief, tissue was cut into ≈20 mg pieces and incubated (10 g tissue/25 ml buffer) in Krebs-Ringer phosphate (KRP buffer, pH 7.4) containing 4% bovine serum albumin (BSA) and 0.5 mg/ml of Collagenase type 1 for 45 min at 37 °C in a shaking water bath. The isolated adipocytes were collected through a 250 μm nylon mesh filter and were washed 3-4 times with 1% KRP-BSA washing buffer. The stromal vascular fraction (SVF) in the washing buffer was collected by 500 × g centrifuge at 4 °C for 10 min. Cells were then homogenized in lysis buffer (0.32 M sucrose, 25 mM KCl, 5 mM MgCl2, 0.1 mM EDTA, 10 mM Tris-HCl pH 7.5, 0.005% NP-40, 1 mM DTT) transferred to ultracentrifuge tubes, layered onto a sucrose cushion solution (1.8 M sucrose, 25 mM KCl, 5 mM MgCl2, 0.1 mM EDTA, 10 mM Tris-HCl pH 7.5, 1 mM DTT) and centrifuged at 106,750 × g for 1 h at 4°C to isolate nuclei.

Bladder epithelium

1. Obtain the surgical specimen.
2. Rinse the specimen in PBS solution.
3. Incubate the bladder in PBS containing 1 mg/ml Collagenase P solution at 37°C during 60 min.
4. Transfer the urothelium to a petri dish and scrub the surface gently with rubber policeman several times. Wash the surface with PBS.
5. Collect the medium to 50ml tube and filter through 100mm cell strainer and wash with PBS.
6. Centrifuge at 1,400 rpm for 10 min.
7. Optional: in the cell pellet is red, resuspend cell in 5ml RBC lysis buffer for 5min on ice. Fill the tube with PBS BSA 0.5% (up to 10-15ml).
8. Filter through 40mm cell strainer and centrifuge at 1,400 rpm for 5 min (if solution cloudy enough count before centrifugation).
9. Resuspend pellet in PBS BSA 0.5% (vol acc pellet).
10. Count cells.
11. Proceed to stain for Flow cytometry sorting (Table - Antibodies for Flow Cytometry).

Blood Granulocytes

1. Venous blood was collected with EDTA (final concentration 1.5-2.0 mM) as anticoagulant
2. Using a Pasteur pipette, 3ml of blood was carefully layered over 3 ml of Polymorphprep (Milteniy Biotec) in a 15 ml centrifuge tube.
3. The prep was centrifuged at 500g for 30-35 min at RT (without brake, Acceleration: 0).
4. The plasma was removed and mononuclear cells (upper band of cells) and the lower band of PMNs (Polymorphonuclear leukocytes, including granulocytes) were harvested separately.
5. An aliquot of Hepes-buffered saline (0.85% (w/v) NaCl, 10 mM Hepes-NaOH, pH 7.4) was diluted with an equal volume of water and 1 vol. of this half-concentration saline was mixed with the PMN suspension. Mononuclear cells (including lymphocytes and monocytes) were mixed with mononuclear cells obtained from the Lymphoprep gradient (see isolation of human mononuclear cells, Lymphocytes and Mononcytes).

6. The PMNs were harvested by centrifugation at 400g for 10 min and resuspended in 0.85% (w/v) NaCl, 10 mM Hepes-NaOH, pH 7.4.
7. To remove any residual erythrocyte contamination of the granulocytes, the cell pellet was resuspended in 3 ml of 1X RBC lysis solution (see recipe below) and incubated at 37°C for 10 min.
8. 3ml PBS, 0.5% BSA was added to the tube and cells were harvested by centrifugation (300g, 5 min) at 4ºC.
9. Pellet was resuspended in 1ml PBS, BSA 0.5%, kept on ice and counted with Trypan Blue in a Bio-Rad automated TC20 Cell Counter.

Breast basal and luminal epithelium

Day 1

Prepare:

Table 1: **DMEM/F12 complete medium:**

	Stock concentration	Final concentration	Add to 100ml sol
FBS	100%	5%	5ml
Insulin	5mg/ml	10mg/ml	1mg (200ml)
hEGF	100mg/ml	10ng/ml	1mg (10ml)
Hydrocortisone	0.5mg/ml	500ng/ml	50mg (100ml)
Glutamine	200mM	2mM	1ml
Pen/Strept	50,000 U/ml Pen 50 mg/ml Strept	100units (Pen) 100mg (Strep)	1ml

Table 2: **Complete Medium + enzymes** (Fresh, prior to dissociation):

	Stock concentration	Final concentration	dilution
Collagenase Type IV	powder	200 units/ml	2.5mg/ml
Hyaluronidase	12500 units/ml	100 units/ml	1:100
DNase I	10mg/ml	10ng/ml	1:1000

1. Obtain breast tissue sample.
2. Wash in PBS.
3. Measure and weight tissue.
4. Transfer to Xml DMEM/F12 complete medium (without enzymes) according table 1 [X=3 times tissue volume].
5. Mince tissue into 3-4 mm pieces.
6. Keep at 4°C until end of the day.
7. Add enzymes according Table 2.
8. Place tube in a shaker at 80 rpm, 37°C overnight.

Day 2

Prepare:

- PBS 2% FBS
- Trypsin B
- DMEM 10% FBS
- RBC lysis buffer x1
- PBS 0.5% BSA

1. Mix the solution - up and down with 10ml pipette- until it became homogeneous.
2. Centrifuge 3 times at 700 RPM 30sec at RT 700 RPM (do not remove supernatant after each centrifugation, only after the last centrifugation step).
3. Transfer all the content without the fat (with a pipette at bottom of tube) to a new 15 ml tube.
4. Add to the supernatant 8 ml PBS 2% FBS.
5. Centrifuge 5 min at 1500 RPM, RT.
6. Resuspend pellet with 7 ml PBS 2% FBS.
7. Centrifuge 5 min, 1500 RPM, RT.
8. Remove supernatant.
9. Add 1 ml trypsin (prewarmed at 37°C).
10. Pipette up and down 3 min with 200ul pipettor, while keeping the tube at 37°C.
11. Stop trypsin by adding 6 ml DMEM 10% FBS.
12. Centrifuge 5 min at 1500 RPM, RT.
13. Remove supernatant (by vacuum).
14. Optional: if the cell pellet is red, resuspend cell in 5ml RBC lysis buffer x1 and incubate for 10min at RT. Fill the tube with PBS 0.5% BSA (up to 10-15ml).
15. Filter cells through 40mm cell strainer.
16. Centrifuge 5 min at 1500 RPM, RT.
17. Resuspend cell pellet in 0.5ml PBS 0.5% BSA.
18. Count cells.
19. Proceed to stain for Flow cytometry sorting (Table - Antibodies for Flow Cytometry).

Cardiomyocytes (Nuclei)

All following steps should be performed on ice. Dissect the left ventricle from fresh or snap-frozen heart with a scalpel. Note, this protocol is optimized for mouse heart but can also be adapted to rat or human heart. Alternatively, use up to 1 g of heart tissue from a different species

Prepare:

- EZ Nuclei lysis buffer in ice
- scalpel + petri dish + PBS
- Refrigerated centrifuge (4 °C)
- Probe homogenizer (us)
- Glass douncer (50 ml) [from Cedar lab]
- 100 μm , 70 μm, 40 μm nylon mesh cell strainer
- 50 and 15 ml tubes
- Filter strainer (before FACS)

1. Trim the specimen into small cubicles with a scalpel.
2. Transfer the tissue pieces into a 50 ml Falcon tube filled with 15 ml of lysis buffer (ice-cold Nuclei EZ lysis buffer).
3. Homogenize the heart tissue with a [Hand Held Homogenizer] 24,000 rpm for 10" . If there are remaining tissue, homogenize for other 10 seconds and transfer the homogenate to a new 50ml tube, then add other 15ml lysis buffer to the remaining tissue and homogenize again. Dilute the homogenate with equal volume of lysis buffer to 30ml.
4. Use a glass douncer (50 ml) [from Cedar lab] to further homogenize the tissue and free the nuclei. Perform eight strokes with a large clearance pestle.

5. Pass the crude nuclei isolate through a 100 µm and 70 µm nylon mesh cell strainer (BD Biosciences), consecutively.
6. Spin down the crude nuclei isolate in a refrigerated centrifuge (4°C) at 700 x g for 10 min.
7. Remove the supernatant carefully by inverting the tubes and wipe the inside of the tube with paper towel. Be careful not to disturb the nuclei pellet.
8. Resuspend with 4ml EZ lysis buffer (Add 1 ml cold Nuclei EZ lysis buffer and mix by pipetting gently with a 1ml tip to completely suspend nuclei pellet. Add the remaining 3 ml of Nuclei EZ lysis buffer, mix well and set on ice for 5 minutes).
9. Collect washed nuclei by centrifugation as in step 6. Carefully aspirate the clear supernatant and set the nuclei pellet on ice.
10. Wash, Resuspend in 4ml PBS. Filter through 40 µm cell strainer to remove clumps and improve purity.
11. Count nuclei.
12. Collect washed nuclei by centrifugation as in step 6.

Nuclei fixation and permeabilization:

13. Fixation in 200ul PFA 1% (rotate for 10 min at RT).
14. Quench by adding 8ul of 2.5M glycine (final concentration 100mM), rotate for 5min at RT.
15. Centrifuge for 5 min 4000 RPM 4°C.
16. Wash in PBS and Centrifuge.
17. Permeabilize in wash solution (PBS,1% BSA, 0.1%Saponin) and rotate for 15min at RT.
18. Centrifuge for 5 min 4000 RPM 4°C.
19. Proceed to stain (in wash solution) for Flow cytometry sorting (Table - Antibodies for Flow Cytometry).

Colon epithelium, Small intestine epithelium, Gall-bladder epithelium, Colon macrophages

Day 1

Prepare:

- ice-cold HBSS (Hank's buffer)
- 0.15% DTT in HBSS
- PBS 10% FBS

1. Obtain the surgical specimen.
2. Place tissue in 10 mL of ice-cold HBSS in a Petri- dish.
3. Cut between the mucosal and the muscle layers with fine curved iris scissors.
4. Put the mucosal strips in a 100 mm Petri dish containing HBSS.
5. After complete removal of the mucosa, rinse strip thoroughly in a Petri dish containing fresh HBSS and transfer them to a 50ml tube containing 50 mL of HBSS, 0.15% dithiothreitol (75mg/50ml). Split into 2 tubes for better shaking.
6. Place tubes on an orbital plate at room temperature, put lid on and set speed at approx 0.30g for 30 min to dissolve residual mucus and free additional debris. At the end of the stirring period, the solution will be slightly cloudy and small floating debris is usually observed.
7. Remove the mucosal strips and the stirring bar, rinse them in a Petri dish with fresh HBSS and transfer them to a new tube with 50 mL.

8. At this step it's possible to keep at 4°C (step 9) or to continue (step 11).
9. Add PBS 10% FBS and keep at 4°C ON.

Day 2

Prepare:

- warmed (37°C) PBS 20mM ph7.2 EDTA
- cold PBS
- Tryp LE (Gibco)

1. Remove the PBS.
2. Add 10 ml pre-warmed (37°C) PBS 20mM ph7.2 EDTA.
3. Put it in warm bath for 10 min at 37°C and mix every 2 min.
4. Put it on ice to settle then remove the PBS/EDTA.
5. Add 10 ml cold PBS and then shake vigorously (at least 10 times).
6. Settle on ice.
7. Transfer the supernatant to a new 50ml tube.
8. Repeat steps (14-16) , check under microscope and decide when to stop the shaking. Under the microscope you would see crypts structures.
9. Centrifuge the crypts: 1000 rpm 7 min at 4°C.
10. Remove the supernatant and resuspend the crypts with 5ml cold PBS and transfer to new 15ml tube.
11. Centrifuge: 200g during 2min at 4°C and remove the supernatant.
12. Re-suspend in 1ml Tryp LE + 0.5mg/ml DNaseI and incubate at 37°C during 10-30 minutes (pipette up and down each 2min) until singles cells were seen under microscope. transfer to 15ml tube.
13. Stop Tryp LE by adding PBS 0.5% BSA (up to 10-15ml).
14. Centrifuge: 300g, 5min at 4°C.
15. Optional: if the cell pellet is red, resuspend cell in 5ml RBC lysis buffer for 10min at RT. Fill the tube with PBS 0.5% BSA (up to 10-15ml).
16. Filter cells through 40mm cell strainer.
17. Centrifuge: 300g, 5min at 4°C.
18. Resuspend in 0.5ml PBS 0.5% BSA.
19. Count cells.
20. Proceed to stain for Flow cytometry sorting (Table - Antibodies for Flow Cytometry).

Cortical Neurons

Cortical neurons (n = 1) were isolated from human occipital cortex by sucrose-gradient centrifugation and labeled with Alexa Fluor 647 conjugate of neuron-specific monoclonal anti-NeuN antibody (A-60) (Millipore, 1:1000). NeuN-positive and negative nuclei were sorted by FACS.

Endometrial epithelium

Prepare:

- Accutase (Life technologies)
- PBS BSA 0.5%

Digestion Media I:

- Collagenase I (3.15mg/ml)
- DNase (40μg/ml)

- DMEM/F12
- FBS 10%
- Pen/strep (Pen 100units, Strep 100mg)

Digestion Media II:

- Collagenase II (0.8mg/ml)
- DNase (40µg/ml)

1. Obtain the surgical specimen.
2. Wash with PBS, mince and incubate for 1 hour at 37ºC in 5ml Digestion media I.
3. Filter the suspension through a 40µm cell strainer.
4. Incubate the remain tissue/clumps (tissue on the filter) for 20min at 37ºC in 5ml Digestion media II.
5. Centrifuge at 1400 rpm for 5 min at 4ºC.
6. Incubate the pellet in 2ml Accutase for 10min at 37ºC.
7. Filter through a 70µm cell strainer, centrifuged at 1400 rpm 5 min 4ºC.
8. If the cell pellet is red, resuspend cells in 5ml cold RBC lysis buffer for 10min at RT. Stop by filling the tube with PBS BSA 0.5% (up to 15ml).
9. Filter through a 40µm cell strainer.
10. Count cells.
11. Proceed to stain for Flow cytometry sorting (Table - Antibodies for Flow Cytometry).

<u>Endothelium from Vein</u>

Prepare:

Collagenase Type II 1mg/ml PBS

- Dispase 1.5mg/ml PBS [2.5U/ml]
- EC medium
- PBS 0.5% BSA
- DNase 10mg/ml

1. Wash the sample with PBS.
2. After removing the connective tissue from the vessel wall with the help of a scissor and a forceps, a longitudinal cut was made along the vein segment with the scissor, and the vascular surface lumen was exposed upwards.
3. Incubate with 20ml Collagenase/Dispase solution during 1hour at 37ºC in shaking incubator at 40 rpm. Put the vascular surface lumen faced-down - the vein was in a glass box- the endothelial cells were down face so all were in the liquid.
4. Add 150ul Dnase I [75ul /10ml Collagenase/Dispase solution] and incubate for another 30min at 37ºC in shaking incubator.
5. Transfer the vein to a petri dish and scrub the surface gently with a rubber policeman several times. Wash the surface with the digestion solution.
6. Transfer the cell suspension to a new tube and put it on ice. Wash again the vascular lumen surface with 50ml EC isolation medium (5ml per 10ml digestion solution) and collect the medium into the 50ml tube.
7. Pass the suspension through 100mm cell strainer.
8. Rinse the filter with additional 5ml cold EC isolation medium.
9. Combine all the tubes and centrifuge at 1500rpm for 5min at RT.
10. If cell pellet is red add 5ml RBC Lysis buffer and incubate for 10min at RT.

11. Fill the tube with PBS BSA 0.5% up to 15ml and filter through 40mm cell strainer, then transfer to 15ml and centrifuge at 1500 for 5min at RT.
12. If cell pellet is still red, repeat steps 10-11 (without filtration).
13. Resuspend cell pellet with 100-200ul PBS BSA 0.5%.
14. Count cells.
15. Proceed to stain for Flow cytometry sorting (Table - Antibodies for Flow Cytometry)

Erythrocyte progenitors

1. Add to 3 ml bone marrow (in EDTA tube) 7 ml PBS.
2. Filtrate through 100 µm cell strainer to remove bone fragments.
3. Lay over 5 ml LymphoPrep.
4. Spin down at 445g for 35min at 20°C.
5. Transfer interphase cells (BM/MNCs) to 50 ml tube.
6. Add 40 ml PBS and spin down at 300g for 10min at 20°C.
7. Resuspend pellet (red) in 3 ml RBC lysis buffer. Incubate during 10min at RT, then add 3 ml PBS 0.5% BSA and spin down at 300g for 10min at 4°C.
8. Resuspend in 1 ml PBS/0.5% BSA.
9. Count cells.
10. Proceed to stain for Flow cytometry sorting (Table - Antibodies for Flow Cytometry).

Esophagus epithelium

Prepare:

- Dispase 0.6-1unit/ml
- PBS BSA 0.5%
- Tryp LE

1. Obtain the surgical specimen.
2. Wash with PBS and incubate for 30 min at 37ºC in 20ml Dispase (8 units/10ml PBS).
3. Separate the epithelial layer form the rest of the tissue, then cut to small pieces and incubate in 3ml Tryp LE in shaking incubator at 37ºC (95rpm) for 15min.
4. Filter the cells through 70µm cell strainer twice and look under the microscope.
5. Centrifuge at 1,200 rpm for 10 min.
6. If the cell pellet is red, resuspend cells in 5ml cold RBC lysis buffer for 10min at RT. Stop by filling the tube with PBS BSA 0.5% (up to 15ml).
7. Count cells.
8. Proceed to stain for Flow cytometry sorting (Table - Antibodies for Flow Cytometry).

Fallopian Tube epithelium

Prepare:

- Cold dissociation medium: 20ml DMEM + 2mg DNase + 30mg Pronase
- PBS 0.5% BSA

Day 1

Wash with PBS twice.

1. Transfer to dissociation medium.
2. Mince the fallopian tube (in case you get the Fimbria, the end of the fallopian tubes) in 20 ml of cold dissociation medium, transfer the minced tissue to a 50-ml centrifuge tube.

3. If you get a different zone, for example, proximal to the uterus, cut the tube along to open it up, cut surrounded tissues from the external part, and Mince into 20 ml of cold dissociation medium.
4. Incubate at 4°C room in an orbital plate during 48hs.

Day 2

1. Invert the tube once or twice to resuspend the minced tissue and then hold the tube upright, allowing bulky pieces to settle to the bottom. Immediately decant the supernatant (containing dissociated epithelial cells) into a second 50-ml centrifuge tube.
2. Add 45 ml of PBS to the first tube containing the minced tissue. Cap the tube and, as in Step 1, invert it to resuspend the tissue pieces. Decant the supernatant into the second 50-ml tube containing pelleted cells.
3. Pellet the decanted epithelial cells by centrifugation at 200g for 5 min at **RT**. Discard the supernatant.
4. To ensure that dissociation enzymes are completely removed from the cells, resuspend the cell pellet in 20 ml of PBS, centrifuge again (200g for 5 min at RT) and discard the supernatant.
5. Look under the microscope to see if cells are in clumps or as singles.
6. In case you don't have single cells, Resuspend in TrypLE, pipette up and down to get single cells (check under microscope).
7. Stop TrypLE by addition of 5ml PBS 0.5% BSA.
8. Filter cells through 100mm cell strainer.
9. Centrifuge at 200g for 5 min at RT.
10. Optional: if pellet is red then resuspend with 5ml RBC lysis buffer and incubate during 10min at RT, then added 5ml PBS 0.5% BSA (if suspension cloudly count now).
11. Filter cells through 40mm cell strainer.
12. Centrifuge at 200g for 5 min at RT.
13. Resuspend with PBS 0.5% BSA and count.
14. Count cells.
15. Proceed to stain for Flow cytometry sorting (Table - Antibodies for Flow Cytometry).

Fibroblasts

- Kanamycin 1:200 (250ul in 50ml PBS)
- Collagenase P (1.5u/mg)
- Dispase: 0.8u/mg- final concentration: 3.2u/ml.

Day 1

1. Obtain the surgical specimen.
2. Wash the specimen in PBS containing kanamycin (100 μg/ml).
3. Mince carefully and transfer into 50ml tube with approximately 10 ml (per1.5g tissue) Collagenase P/ Dispase in DMEM (without FBS).
4. Incubate at 37°C for 45 min.
5. Add 30ml complete medium (DMEM supplemented with 10%FBS/ 1 × penicillin/streptomycin/ Glutamine) and incubate ON at 37 °C in a tissue incubator (not a shaking incubator).

Day 2:

1. Dissociate the tissue by gentle pipette up and down.

2. Filter through 100mm cell strainer.
3. Centrifuge in 1400 RPM 5 min, resuspend pellet 5ml in PBS 0.5% BSA.
4. Centrifuge in 1400 RPM 5 min.
5. Optional: if the cell pellet is red, resuspend cell in 5ml RBC lysis buffer for 5 min on ice. Fill the tube with PBS BSA 0.5% (up to 10-15ml).
6. Filter through 40mm cell strainer and centrifuge at 1,400 rpm for 5 min.
7. Resuspend pellet in PBS BSA 0.5% (vol acc pellet).
8. Count cells.
9. Proceed to stain for Flow cytometry sorting (Table - Antibodies for Flow Cytometry).

Gastric antrum, body or fundus epithelium

Day 1

Prepare:

- ice-cold HBSS
- 0.15% DTT in HBSS
- PBS 10%FBS

1. Obtain the surgical specimen.
2. Place tissue in 10 mL of ice-cold HBSS in a Petri- dish.
3. Cut between the mucosal and the muscle layers with fine curved iris scissors.
4. Put the mucosal strips in a 100 mm Petri dish containing HBSS.
5. After complete removal of the mucosa, rinse strip thoroughly in a Petri dish containing fresh HBSS, cut the mucosa to 1x1 cm pieces and transfer them to a 50ml tube containing 50ml HBSS, 0.15% DTT (75mg/50ml). Split in to 2 tubes for better shaking.
6. Place tubes on orbital plate at RT, put lid on and set speed at approx. 0.30g for 30min to dissolve residual mucus and free additional debris. At the end of the stirring period, the solution will be slightly cloudy and small fluting debris is usually observed.
7. Remove the mucosal strips and the stirring bar, rinse them in a Petri dish with fresh HBSS and transfer them to a new tube with 50ml PBS 10%FBS.
8. Keep at 4°C ON

Day 2

Prepare:

- Enzymatic digestion solution:
    - 20 ml of prewarmed DMEM (37°C)
    - 10 mM HEPES Buffer
    - 1 mg/mL Collagenase Type I
    - 2 mg/mL BSA
- pre-heated DMEM/F12
- TrypLE
- DNase (10mg/ml)

1. Remove the PBS.
2. Add enzymatic digestion solution.
3. Incubate at 37°C in a shaking incubator (60 rpm) for 45 min. After the tissue has been incubated, remove 50 µL of incubation media and check visually for dissociated glands. If glands are not dense or have not separated from the tissue, shake gently 2-3 times.

4. Immediately following incubation, add 20 mL pre-heated DMEM/F12 to incubation media using a sterile serological pipette or by pouring directly into the incubation mixture.
5. Filter cells through 100mm.
6. Transfer the mixture into 15 mL cell culture test tubes. Centrifuge for 5 min at 1100 rpm at 4 °C (if the supernatant is still cloudy, centrifuge again).
7. Resuspend cell pellet in 1ml Tryp LE + 0.5mg/ml DNaseI [adjust volume of Tryp LE and DNaseI according to cell pellet] Pipette up and down until single cells are visible under microscope.
8. Add PBS (up to 10ml) at room temp to Stop the TrypLE.
9. Filter cells through 40mm cell strainer.
10. Centrifuge at 300g, 5min at 4°C.
11. Optional: if the cell pellet is red, resuspend cell in 5ml RBC lysis buffer for 10 min at RT. Fill the tube with PBS 0.5% BSA (up to 10-15ml) and centrifuge at 300g, 5min at 4°C.
12. Resuspend cell in volume according to cell pellet.
13. Count cells.
14. Proceed to stain for Flow cytometry sorting (Table - Antibodies for Flow Cytometry).

Hepatocytes

Prepare:

Buffer A (4ml/500mg tissue)

Final Conc	Stock	For 20ml (in DDW)
250 mM sucrose	1M	5ml
5 mM MgCl2	1M	100ml
10 mM Tris–HCl [pH 7.4]	1M	200ml

Buffer B (9 volumes/pellet)

Final Conc	Stock	For 1ml (in DDW)
2M sucrose	2M	1ml
1 mM MgCl2	1M	1ml
10 mM Tris–HCl [pH 7.4]	1M	10ml

1. Clean the tissue from fat and connective tissue.
2. Weight the cleaned tissue and cut it to small pieces.
3. Mash liver pieces through a 40μm cell strainer with the plunger from a 5-mL syringe, then 1ml syringe into a plastic Petri dish on ice with 4 mL of ice-cold Buffer A.
4. Centrifuge the mass of disrupted cells at 600g for 10 min at 4°C.
5. Gently resuspend the mass of disrupted cells in 14 mL of ice-cold Buffer A and centrifuged as in step 3. Discard the supernatant.
6. Resuspend the crude nuclei pellet in 9 volumes of ice cold Buffer B mix well and distribute into eppendorf tubes of 1.5 mL, centrifuged at 16,000g at 4°C for 30 min.
7. We need to decide what to do with this: [The crude nuclei were separated into two layers. The upper layer, which was brownish and sticky, was deposited at the surface of the buffer, whereas the white pellet of isolated nuclei was on the bottom of the tube].
8. The tube was inverted and pushed gently against a paper towel, removing most of the upper layer by absorption onto the towel. Materials adhering to the tube walls were wiped off with cotton swabs.

9. Resuspend the pellet of isolated nuclei in PBS (500ul and centrifuge at 700g for 10min). [Look at the microscope, if the percentage of nuclei is low, centrifuge again as in step 4].
10. Filter through 40μm cell strainer, let the solution goes without forcing it.
11. Stain with nuclear dye or fix.

Kidney Tubular and glomerular epithelium

Prepare:

- 40 ml HBSS 0.1% BSA
- 0.1% Collagenase IV in 20ml HBSS with 0.1% BSA (20ml solution / 0.7 gr tissue)
- 0.6% Collagenase IV in 20ml HBSS with 0.1% BSA (20ml solution / 0.7 gr tissue)
- HBSS 5% FBS (20ml)

1. Obtain the surgical specimen.
2. Place the tissue in 20 ml HBSS with 0.1% BSA and 0.1% Collagenase IV finely minced with scalpels in a glass petri dish.
3. Incubate at 37°C in a shaking incubator (80 rpm) for 30 min.
4. Pipette up and down and pass through a 100mm cell strainer (to separate between glomerulus (the chunk) and the tubular (the sup).
5. Wash the100mm cell strainer with HBSS 5% FBS and centrifuge at 1,200 rpm for 4 min in 4°C. [this is the tubular fraction]. Resuspend the pellet in PBS 0.5% BSA. Go to step 8.
6. Take the chunk (glomerulus) from the filter, put in 20 ml HBSS with 0.1% BSA and 0.6% Collagenase IV and incubate at 37°C in a shaking incubator (80rpm) for 30 min.
7. Pipette up and down and pass through a 100mm filter. Wash the filter with HBSS 5% FBS and centrifuge at 1,200 rpm for 4 min in 4°C. [this is the glomerular fraction]. Resuspend the pellet in PBS 0.5% BSA. Go to step 8.
8. Centrifuge at 1,500 rpm for 4 min.
9. The pellet was enzymatically and mechanically dissociated into single cells with 1ml Tryp LE + 0.5mg/ml DNaseI.
10. Stop Tryp LE by filling the tube with PBS BSA 0.5% up to 15ml. Passed through a 70mm cell strainer to remove debris and centrifuge at 1,500 rpm for 4 min.
11. Optional: if the cell pellet is red, resuspend cells in 5ml RBC lysis buffer (diluted fresh) for 5 min **on ice**. Fill the tube with PBS BSA 0.5% up to 15ml and filter through a 40mm cell strainer to remove remaining cell aggregates and debris.
12. Count cells.
13. Proceed to stain for Flow cytometry sorting (Table - Antibodies for Flow Cytometry).

Larynx and Pharynx epithelium

Day 1

1. Obtain the surgical specimen.
2. Cut the tissue into small pieces and incubate in pronase 0.15% in DMEM-F/12.
3. Keep at 4°C over night.

Day 2

Shake the tube several times and check under the microscope.

1. Add 10mg Dispase into 11 ml medium from step 2 and incubate 15 min at 37°C in a shaking incubator.
2. Collect the sup by pipette and pass it through 100mm cell strainer.
3. Centrifuge at 1,200 rpm for 5 min.

4. Add to the pellet 3ml Tryp LE and 200ml DNase (10mg/ml), incubate and pipette, for 3 min, cheek under the microscope and if you see single cells stop the reaction by adding PBS BSA 0.5%.
5. Centrifuge at 1,200 rpm for 5 min.
6. If the cell pellet is red, resuspend cells in 5ml cold RBC lysis buffer for 10min in RT. Fill the tube with PBS BSA 0.5% (up to 15ml).
7. Count cells.
8. Proceed to stain for Flow cytometry sorting (Table - Antibodies for Flow Cytometry).

Lung alveolar epithelium, bronchial epithelium, interstitial lung macrophages, lung alveolar macrophages.

Day 1

Prepare:

- BBS buffer: Hank's buffer solution, 20mM Hepes ph 7.3

1. Wash the tissue in BBS buffer and cut it to small pieces with scissors (on ice), transfer all to 50 ml tube.
2. Centrifuge at 1200 rpm 5min 4°C. After centrifugation, continue with both, floating pieces and precipitates pieces.
3. Wash the tissue with BBS buffer and centrifuge at 1200 rpm 5min 4°C 3 times.
4. Keep tissue in BSS buffer ON at 4°C.

Day 2

Prepare:

- Digestion solution: 5U/ml Elastase (Worthington) dissolved in 3ml Trypsin B (Biological Industries) and 10ug DNaseI (Sigma)
- DMEM
- FBS (heat inactivated)
- RBC lysis buffer x1

1. Filter the tissue suspension through 100mm filter.
2. Transfer the remaining tissue on the filter) to a new 50 ml tube.
3. Add digestion solution and incubate for 3 hours at 37°C in a shaking incubator (90 rpm).
4. Add 2mg DNaseI and shake for 15 minutes at 37°C.
5. Add 30ml DMEM, 20% FBS, 0.4mg DNaseI to stop digestion.
6. Filtrate all the volume through 100mm cell strainer, use a syringe plunger to disrupt remaining tissue pieces through the strainer.
7. Filtrate through 70mm and 40mm cell strainer filter.
8. Centrifuge at 400g 10min 4°C.
9. Throw out supernatant (if there is a visible pellet, by inverting the tubes, if not, so gently with a pipette).
10. Optional: if the cell pellet is red, resuspend cell pellet with 5ml RBC lysis buffer. Incubate during 10 min at RT. Fill the tube with PBS 0.5% BSA (up to 10-15ml).
11. Filter cells through 40mm cell strainer.
12. Centrifuge at 400g 10min 4°C.
13. Resuspend cell pellet in 0.5ml PBS 0.5% BSA.
14. Count cells.
15. Proceed to stain for Flow cytometry sorting (Table - Antibodies for Flow Cytometry).

Mononuclear Cells (Lymphocytes and Monocytes)

1. Venous blood was collected with EDTA (final concentration 1.5-2.0 mM) as anticoagulant.
2. 3ml of blood was diluted with an equal volume of saline (0.85% NaCl).
3. Using a Pasteur pipette, 6ml of diluted blood was carefully layered over 3 ml of Lymphoprep (Milteniy Biotec) in a 15 ml centrifuge tube.
4. The prep was centrifuged at 800g for 20 min at room temperature (without brake, Acceleration: 0).
5. After centrifugation the mononuclear cells form a distinct band at the sample/medium interface. These cells were removed using a Pasteur pipette mixed with mononuclear cells obtained from the Polymorphprep gradient and diluted with an equal volume of 0.85% NaCl and centrifuged for 10 min at 300g.
6. If required erythrocytes were removed with RBC Lysis solution (see **#7 Isolation of Granulocytes**).
7. Pellet was resuspended in 1ml PBS, BSA 0.5%, kept on ice and counted with Trypan Blue in a Bio-Rad automated TC20 Cell Counter.
8. Cells were stained for Flow cytometry sorting (Table - Antibodies for Flow Cytometry).

Ovary epithelium

Prepare:

- HBSS
- Collagenase Type I (300U/ml)
- Medium 199

1. Obtain the surgical specimen.
2. Rinse the specimen in Hank's solution.
3. Incubate the tissue in 20ml medium 199 containing Collagenase Type I (300U/ml) at 37°C during 40 min.
4. Transfer the tissue into a 50 ml centrifuge tube containing 10 ml of medium 199.
5. Gently pipet and vortex the medium from step 3 (the one was incubated with the tissue) at 30 rpm for 60 sec.
6. Put the tissue in a 60 mm plastic culture dish (Corning) with 10 ml of the same medium (from step 5), scrap the ovarian surface with a no. 11 surgical blade.
7. Collect cells by centrifugation at 1,400 rpm for 5 min.
8. Resuspend pellet in 3 ml PBS 0.5% BSA and allowed to stand for 15 min at room temperature.
9. Add PBS (10ml) and collect cells by centrifugation at 1,400 rpm for 5 min.
10. Incubate in TrypLE and DNase (50ml/1ml) at 37°C for 5-20min, look under microscope until cell clumps become single cells.
11. Stop TrypLE by addition of 5 ml PBS 0.5% BSA, filtrate through 70mm cell strainer and centrifuge at 1,400 rpm for 5 min.
12. Optional: in the cell pellet is red, resuspend cell in 5ml RBC lysis buffer for 5 min on ice. Fill the tube with PBS BSA 0.5% up to 15ml.
13. Filter through 40mm cell strainer and centrifuge at 1,400 rpm for 5 min.
14. Rhesus d pellet in PBS BSA 0.5% (vol acc pellet).
15. Count cells.
16. Proceed to stain for Flow cytometry sorting (Table - Antibodies for Flow Cytometry).

Pancreas islet/duct/acinar cells

Day 1

1. Obtain the surgical specimen.
2. Incubate over weekend in PIM's at 37 °C incubator (PIM medium is purchased from PRODO laboratories Inc.)

Day 2

1. Collect all the islet from the petri dish in to 50ml tube and centrifuge in 200g 4 °C for 5 min.
2. resuspend pellet 10ml in PBS and centrifuge again in 200g 4 °c for 5 min.
3. Dissociate pellet in 5ml accumax and 1:100 DNase (50ml from 10mg/ml), split into 5X eppendorf tubes (1ml/tube) for a better Dissociation and incubate in 37 °C 12min.
4. Pipet 10 times and add same volume of PBS FBS 20% (5ml total) to quench accumax.
5. Filter through 70mm cell strainer, centrifuge again 500g 4 °C for 5 min.
6. Wash with PBS BSA 0.5%, Count cells.
7. Stain in wash solution.

Prostate epithelium

Day 1

Prepare:

- cold HBSS with 1% BSA
- cold PBS
- Tryp LE
- dissociation medium (10ml dissociation medium/gr tissue)
- HBSS
- 0.8-1.5mg/ml Collagenase Type II
- 0.2mg/ml DNaseI
- 10mg/ml Pen/strep

1. Weigh tissue and transfer it to 100mm plate with cold HBSS with 1% BSA.
2. Use scissors and forceps to cut tissue into small pieces (enough small to be passed through a 10ml pipette).
3. Transfer tissue pieces to a conical tube.
4. Centrifuge at 1800rpm for 5 min at 4°C.
5. Aspirate supernatant and resuspend in 30ml cold PBS. Repeat wash.
6. Add dissociation medium (ratio 10ml/gr of tissue).
7. Incubate ON overnight at 37°C in a shaking incubator.

Day 2

Prepare:

- cold PBS
- TrypLE
- DMEM 10% FBS
- DNaseI 10mg/ml
- RBC lysis buffer

1. Wash 2X in 25ml cold PBS (in the last wash pipette up and down with 10ml pipette).

2. Centrifuge at 1800rpm for 5 min at 4°C.
3. Resuspend cells in 5 ml TrypLE and incubate for 5 min on 37°C (shaking incubator).
4. Add to TrypLE: 15 ml DMEM + 10% FBS + 1mg/ml DNaseI.
5. Centrifuge at 1800rpm for 5 min at 4°C.
6. Resuspend in 10ml DMEM + 10% FBS and pass through 19-G needle/10ml syringe 2–5X. Repeat with 21-G. (Note: if tissue cannot be passed through 18-G needle, first proceed to step 16, forcing through needles decreases viability).
7. Filter cells/media through 100µm cell strainer and wash conical tube with additional 10 ml DMEM + 10% FBS.
8. Centrifuge at 1800rpm for 5 min at 4°C.
9. Resuspend cell in 5ml RBC lysis buffer for 5min on ice. Fill the tube with PBS 0.5% BSA (up to 10-15ml).
10. Filter through 40µm cell strainer.
11. Centrifuge at 1500rpm for 7 min at 4°C.
12. Resuspend cell in PBS 0.5% BSA in volume according to cell pellet.
13. Count cells.
14. Optional: if fraction of live cells is above 30%, go to step 36, otherwise proceed to step 22.

<u>Depletion of dead cells using magnetic beads Dead cell Kit (Miltenyi)</u>

<u>Magnetic Separation:</u>
BBK Buffer: binding buffer, part of the kit, concentrated x20, to be diluted in DDW just before use

15. Centrifuge cells at 300g, 10min, 4°C.
16. Resuspend cells in 100ml [up to 1x10^7 cells] of DCK micro beads (dead cell kit).
17. Incubate 15 min at RT.
18. Activation of the column (LS column) with 3ml BBK buffer.
19. Add 1ml of BBK buffer to cell suspension with beads.
20. Apply suspension onto the column and collect the effluent (live cell fraction) into a new 15 ml tube.
21. Wait until all the suspension passes the column.
22. Wash x3 with 3ml BBK buffer.
23. Centrifuge effluent at 300g, 10 min, 4°C.
24. Wash with 5ml PBS 0.5% BSA.
25. Centrifuge at 300g, 10 min, 4°C.
26. Resuspend cell in PBS 0.5% BSA in volume according to cell pellet.
27. Count cells.
28. Proceed to stain for Flow cytometry sorting (Table - Antibodies for Flow Cytometry).

<u>Thyroid epithelium</u>
1. Obtain the surgical specimen.
2. Wash the specimen in Hanks solution containing 5% Pen/Strept.
3. Mince carefully in the Collagenase IV (100 IU/ml) and Dispase (1 mg/ml) in 10 ml HBS at 37°C for 1 hour and every 15 min gently pipette.
4. Stop digestion by adding FBS to the mixture (final concentration 0.5%). Pass the sample through 100mm cell strainer.

5. If there is a remaining tissue digest in a new enzymatic mixture.
6. Check under the microscope: if there are no single cells add TrypLE according to the pellet.
7. If there are single cells continue to step 8.
8. Centrifuge in 1400 RPM 5 min.
9. Optional: if the cell pellet is red, resuspend cell in 5ml RBC lysis buffer for 5min on ice. Fill the tube with PBS BSA 0.5% (up to 10-15ml).
10. Filter through 40mm cell strainer and centrifuge at 1,400 rpm for 5 min.
11. Resuspend pellet in PBS BSA 0.5% (vol acc pellet).
12. Count cells.
13. Proceed to stain for Flow cytometry sorting (Table - Antibodies for Flow Cytometry).

Tongue epithelium

Prepare:

- Enzyme mixture: Mix Collagenase Type 1 (550 U/mL, Worthington) and Elastase (10 U/mL, Worthington) in PBS
- DMEM medium
- FBS
- Pen/strep (Pen 100 units, Strep 100mg)

Day 1

1. Wash the tissue with PBS.
2. Separate the epithelial layer from the muscle layer.
3. Cut the tissue into small pieces.
4. Keep the tissue ON at 4°C in DMEM, FBS 10% and Pen/Strep.

Day 2

1. Wash the tissue in PBS.
2. Incubate the tissue for 1hour in a shaking incubator at 37°C with enzyme mixture.
3. Pipette up and down with 1ml pipettor multiple times.
4. Filter the cells through 100µM cell strainer and centrifuge 5min at 1200 RPM.
5. Optional: If the pellet is red resuspend in 5ml of 1X RBC lysis buffer for 10 min at RT with occasional shaking.
6. Add up to 15 ml with cold PBS 0.5% BSA and filter through 40µm cell strainer.
7. Count cells.
8. Proceed to stain for Flow cytometry sorting (Table - Antibodies for Flow Cytometry).

Tonsil epithelium

Day 1

Prepare:

- DMEM
- Pen/strep (Pen 100 units, Strep 100mg)
- FBS
- Collagenase II (1mg/ml)
- Dispase (0.5mg/ml)

1. Obtain the surgical specimen.
2. Wash with PBS.

3. Place the tissue in 10 ml DMEM with 5% FBS and Pen/strep, finely mince with scalpels in a glass petri dish on ice until the next step.
4. At the end of the day add enzymes Collagenase II and Dispase dissolved in 10ml DMEM 5% FBS.
5. Incubate ON at 37°C in a shaking incubator (80rpm).

Day 2

Prepare:
- DMEM FBS 2%
- DNaseI (10mg/ml)
- PBS BSA 0.5%
- CD45 MicroBeads, human

1. Mix the suspension - up and down with 10ml pipette- until it becomes homogeneous.
2. Wash the resultant cell suspension with DMEM FBS 2% 3 times by centrifugation at 1,200 rpm for 4 min in 4ºC.
3. Dissociate the pellet into single cells with 1ml Tryp LE + 0.5mg/ml DNaseI. Pipet up and down and look under a microscope until you get single cells.
4. Stop the dissociation by adding PBS BSA 0.5% up to 50ml. Filter through a 100µm cell strainer to remove debris and centrifuge at 1,500 rpm for 4 min.
5. Optional: in the cell pellet is red, resuspend cells in 5ml RBC lysis buffer for 5min on ice. Then fill the tube with PBS BSA 0.5% up to 15ml.
6. Filter through a 40µm cell strainer to remove remaining cell aggregates and debris.
7. Deplete the leukocyte population using human CD45 MicroBeads according manufacture protocol.
8. Count cells.
9. Proceed to stain for Flow cytometry sorting (Table - Antibodies for Flow Cytometry).

Appendix - RBC lysis buffer (X10):

- NH_4Cl (ammonium chloride) 8.02mg
- $NaHCO_3$ (sodium bicarbonate) 0.84mg
- EDTA (disodium) 0.37mg
- QS to 100ml with Millipore water
- Store at 4°C for six months.

Working solution:

Dilute 10ml 10X concentrate with 90 ml Millipore water. Refrigerate until use

Supplementary Table S18 - Antibodies for Flow Cytometry

Cell type	Positive selection	Negative selection
Bladder epithelium	CD326	CD45, CD31, CD235
Breast basal epithelium	CD326, CD49	CD45, CD31, CD235
Breast luminal epithelium	CD326	CD45, CD31, CD235
Cardiomyocytes (Nuclei)	Nuclei stain, Nkx2.5	
Colon epithelium	CD326	CD45, CD31, CD235
Colon macrophages	CD14 , CD33	
Cortical Neurons (Nuclei)	NeuN	
Endometrial epithelium	CD326	CD45, CD31, CD235
Endothelium from Vein	CD105	CD45
Erythrocyte progenitors	CD235, CD71	CD45
Esophagus epithelium	CD326	CD45, CD31, CD235
Fallopian Tube epithelium	CD326	CD45, CD31, CD235
Fibroblasts	Vimentin	CD45, CD105, CD235, CD326
Gall-bladder epithelium	CD326	CD45, CD31, CD235
Gastric antrum, body or fundus epithelium:	CD326	CD45, CD31, CD235
Hepatocytes	Nuclei stain (Hoechst), 4N nuclei	
Interstitial lung macrophages	CD14, CD169, CD206	
Kidney tubular epithelium	CD326	CD45, CD31, CD235
Kidney glomerular epithelium	CD326, Nephrin	CD45, CD31, CD235
Larynx epithelium	CD326	CD45, CD31, CD235
Lung alveolar epithelium	CD326	CD45, CD31, CD235
Lung alveolar macrophages	CD14, CD169	CD206
Lung bronchial epithelium	CD326	CD45, CD31, CD235
Ovary epithelium	CD326	CD45, CD31, CD235
Pancreas-Beta cells	C-peptide	Glucagon/ Somatostatin
Pancreas-Alpha cells	Glucagon	C-peptide/Somatostatin
Pancreas-Delta cells	Somatostatin	C-peptide/Somatostatin
Pancreas-Acinar cells	HIC03B3 (Grompe Lab)	DHIC5-4D9 (Grompe Lab)
Pancreas-Duct cells	DHIC5-4D9 (Grompe Lab)	HIC03B3 (Grompe Lab)
Pharynx epithelium	CD326	CD45, CD31, CD235
Prostate epithelium	CD326	CD45, CD31, CD235
Small intestine epithelium	CD326	CD45, CD31, CD235
Thyroid epithelium	CD326	CD45, CD31, CD235
Tongue epithelium	CD326	CD45, CD31, CD235
Tonsil epithelium	CD326	CD45, CD31, CD235

Blood cells	Positive selection	Negative selection
B cells	CD45, CD19	CD3
Blood granulocytes	CD45, CD14low, CD66abce	CD3, CD56
memory B-cells	CD19, CD27	
Monocytes	CD45, CD14	CD3, CD56
Naive T cells CD4	CD4, CD197, CD45RA	CD45RO

Naive T cells CD8	CD8, CD197, CD45RA	CD45RO
NK cells	CD45, CD56	CD3, CD14
T (CD3+) cells	CD45, CD3	
T Central memory CD4	CD4, CD197, CD45RO	CD45RA
T cytotoxic (CD8+) cells	CD45, CD3, CD8	
T effector cell CD8	CD8, CD45RA	CD197, CD45RO
T Effector memory CD4	CD4, CD45RO	CD197, CD45RA
T Effector memory CD8	CD8, CD45RO	CD197, CD45RA
T helper(CD4+) cells	CD45, CD3, CD4	

**Antibodies**	**Source**	**ID**
CD105-APC	Miltenyi Biotec	Cat# 130-094-926, RRID:AB_10827601
CD14-APC ef780	Thermo Fisher Scientific	Cat# 47-0149-42, RRID:AB_1834358
CD14-APC V10770	Miltenyi Biotec	Cat# 130-113-706, RRID:AB_2726247
CD169-APC	Thermo Fisher Scientific	Cat# 17-1699-42, RRID:AB_10854878
CD19-VioGreen	Miltenyi Biotec	Cat# 130-114-175, RRID:AB_2726479
CD197-PE	Thermo Fisher Scientific	Cat# 12-1979-42, RRID:AB_10670625
CD206-PE	Thermo Fisher Scientific	Cat# 12-2069-42, RRID:AB_10804655
CD235a-eF450	Thermo Fisher Scientific	Cat# 48-9987-42, RRID:AB_2574141
CD27-APC	Thermo Fisher Scientific	Cat# 17-0279-42, RRID:AB_10671130
CD3-FITC	Miltenyi Biotec	Cat# 130-120-267, RRID:AB_2876969
CD3-FITC	Thermo Fisher Scientific	Cat# 11-0037-42, RRID:AB_2016669
CD31-alexa488	BD Biosciences	Cat# 558068, RRID:AB_647081
CD31-eF450	Thermo Fisher Scientific	Cat# 48-0319-42, RRID:AB_10854276
CD33-APC	Thermo Fisher Scientific	Cat# 17-0338-42, RRID:AB_10667893
CD4-Peap-ef710	Thermo Fisher Scientific	Cat# 46-0041-82, RRID:AB_11150050
CD4-Percp v10700	Miltenyi Biotec	Cat# 130-113-819, RRID:AB_2726335
CD45RA-FITC	Miltenyi Biotec	Cat# 130-113-365, RRID:AB_2726135
CD45RO-APC	BD Biosciences	Cat# 559865, RRID:AB_398673
CD49f-FITC	BD Biosciences	Cat# 555735, RRID:AB_396078
CD66-abce-PE	Miltenyi Biotec	Cat# 130-117-811, RRID:AB_2733834
CD71-FITC	Thermo Fisher Scientific	Cat# 11-0719-42, RRID:AB_1724093
CD8-APC V10770	Miltenyi Biotec	Cat# 130-096-561, RRID:AB_10829313
CD8-APC-eF780	Thermo Fisher Scientific	Cat# 47-0088-42, RRID:AB_1272046
Anti-Nephrin	Abcam	Cat# ab136894, RRID:AB_2894886
Anti-Nkx2.5	Cell Signaling Technology	Cat# 8792, RRID:AB_2797667
EpCAM-APC	Miltenyi Biotec	Cat# 130-113-260, RRID:AB_2726061
EpCAM-PE	Miltenyi Biotec	Cat# 130-111-116, RRID:AB_2657494
CD45-eF450	Thermo Fisher Scientific	Cat# 48-9459-42, RRID:AB_1603240
CD45-PE	Thermo Fisher Scientific	Cat# 12-9459-42, RRID:AB_10718238
CD56-APC	Miltenyi Biotec	Cat# 130-100-698, RRID:AB_2658734
CD56-APC	Thermo Fisher Scientific	Cat# MA1-19462, RRID:AB_1073071
Anti-C-peptide	DSHB	Cat# GN-ID4, RRID:AB_2255626
Anti-Glucagon	Abcam	Cat# ab92517, RRID:AB_10561971
Hoechst	Thermo Fisher Scientific	H1399
Anti-Somatostatin	Abcam	Cat# ab108456, RRID:AB_11158517

Anti-Vimentin-488	BD Biosciences	Cat# 562338, RRID:AB_10896994

Supplementary Table S19 - Reagents used

Reagents	Source	ID
Accumax	Sigma	A7089
Accutase	Life technologies	00-4555-56
Albumin Bovine (BSA)	Tamar	0332-TAM
CD45 MicroBeads	Miltenyi	130-045-801
Collagenase P	Roche	11213865001
Collagenase type I	thermo	17018029
Collagenase type II	thermo	17101015
Collagenase Type IV	Sigma	C5138
Dead cell Kit	Milteniy Biotec	130-090-101
Dispase	GIBCO	17105-041
DMEM	Biological Industries	01-055-1A
DMEM/F12	Biological Industries	01-170-1A
DNase I	Roche	11284932001
Elastase	Worthington	LS002292
EZ Nuclei lysis buffer	Sigma	nuc101-1kt
FBS	Biological Industries	04-001-1A
Glutamine	Biological Industries	03-020-1B
Hank's solution	Biological Industries	02-018-1A
hEGF	PEPROTech	AF-100-15
HEPES Buffer 1M	Biological Industries	03-025-1C
Hyaluronidase	Sigma	H3506
Hydrocortisone	Sigma	H0888
Insulin	Sigma	I1882
Kanamycin	Sigma	K1377
Lymphoprep gradient	Milteniy Biotec	04-03-9391/02 Serumwerk
Medium 199	Biological Industries	01-080-1A
PBS	Biological Industries	02-023-1A
Pen/Strept	Roche	11074440001
PIM medium	PRODO laboratories	no cat number
Polymorphprep	Milteniy Biotec	04-03-9393/01 Serumwerk
Pronase	Roche	165-921-001
Tryp LE	thermo	12604013
Trypan Blue	BIO-RAD	145-0013
Trypsin B	Biological Industries	03-052-1B
