# Supplementary material for: A DNA methylation atlas of normal human cell types
Source: Nature. 2023 Jan 4;613(7943):355–64. doi: 10.1038/s41586-022-05580-6 (PMC9811898; doi:10.1038/s41586-022-05580-6)
Supplement: Supplementary file 2 — Reporting Summary [file 41586_2022_5580_MOESM2_ESM.pdf]

nature portfolio

Corresponding author(s): Yuval Dor, Benjamin Glaser, Tommy Kaplan

Last updated by author(s): Oct 6, 2022

Reporting Summary

Nature Portfolio wishes to improve the reproducibility of the work that we publish. This form provides structure for consistency and transparency in reporting. For further information on Nature Portfolio policies, see our Editorial Policies and the Editorial Policy Checklist.

Statistics

For all statistical analyses, confirm that the following items are present in the figure legend, table legend, main text, or Methods section.

n/a	Confirmed	
☐	☒	The exact sample size (*n*) for each experimental group/condition, given as a discrete number and unit of measurement
☐	☒	A statement on whether measurements were taken from distinct samples or whether the same sample was measured repeatedly
☐	☒	The statistical test(s) used AND whether they are one- or two-sided<br>*Only common tests should be described solely by name; describe more complex techniques in the Methods section.*
☐	☒	A description of all covariates tested
☐	☒	A description of any assumptions or corrections, such as tests of normality and adjustment for multiple comparisons
☐	☒	A full description of the statistical parameters including central tendency (e.g. means) or other basic estimates (e.g. regression coefficient) AND variation (e.g. standard deviation) or associated estimates of uncertainty (e.g. confidence intervals)
☐	☒	For null hypothesis testing, the test statistic (e.g. *F*, *t*, *r*) with confidence intervals, effect sizes, degrees of freedom and *P* value noted<br>*Give P values as exact values whenever suitable.*
☐	☒	For Bayesian analysis, information on the choice of priors and Markov chain Monte Carlo settings
☐	☒	For hierarchical and complex designs, identification of the appropriate level for tests and full reporting of outcomes
☐	☒	Estimates of effect sizes (e.g. Cohen's *d*, Pearson's *r*), indicating how they were calculated

Our web collection on statistics for biologists contains articles on many of the points above.

Software and code

Policy information about availability of computer code

Data collection	no software was used
Data analysis	bedtools (v 2.26.0)<br>bedGraphToBigWig (V 4)<br>deepTools (V 3.4.1)<br>multiBigwigSummary(V 3.4.1)<br>bwa-meth (V 0.2.0)<br>SAMtools (V 1.9)<br>Sambamba (V 0.6.5)<br>wgbstools (V 0.1.0)<br>sklearn KNNImputer (V 0.24.2)<br>scipy (V 1.6.3)<br>ggtree (V 2.2.4)<br>GREAT (V 4.0.4, https://great.stanford.edu/)<br>HOMER findMotifsGenome.pl (V2)<br>wgbstools (v 0.1.0, https://github.com/nloyfer/wgbs_tools)<br><br>Code is available at github.com/nloyfer/wgbs_tools and github.com/nloyfer/UXM_deconv

For manuscripts utilizing custom algorithms or software that are central to the research but not yet described in published literature, software must be made available to editors and reviewers. We strongly encourage code deposition in a community repository (e.g. GitHub). See the Nature Portfolio guidelines for submitting code & software for further information.

Data

Policy information about availability of data

All manuscripts must include a data availability statement. This statement should provide the following information, where applicable:

- Accession codes, unique identifiers, or web links for publicly available datasets
- A description of any restrictions on data availability
- For clinical datasets or third party data, please ensure that the statement adheres to our policy

DNA methylation data is available in bigwig format (position and average methylation across 27,927,160 CpGs), and beta format (a similar wgb tools-compatible binary format) at the GEO, accession GSE186458. Bigwig and beta files for hg38 are also available. Fragment-level information (in pat format, including CpG starting index, methylation pattern of all covered CpGs, and number of fragments with this exact multi-CpG pattern) are also available. Raw fastq can be downloaded upon request to EGA (through the atlas Data Access Committee).

Human research participants

Policy information about studies involving human research participants and Sex and Gender in Research.

Reporting on sex and gender	No gender-related data was collected. Sex data is detailed in Extended Table S1
Population characteristics	We used >200 tissue specimens from consented patients admitted to surgery at Hadassah Medical Center. Detailed information on donors is provided in Extended Table S1.
Recruitment	This is not a population-based study. We are defining tissue-specific methylation patterns that are universally conserved among all individuals. Prospective donors were approached, received an explanation and signed informed consent.
Ethics oversight	Study was approved by the Helsinki committee of the Hadassah Medical Center. Some cells and tissues were obtained through collaborative arrangements (Extended Table S1). These include pancreatic exocrine and liver samples (cadaveric organ donors, n=5) from Prof. Markus Grompe, Oregon Health & Science University. Adipocytes (subcutaneous adipocytes at time of cosmetic surgery following weight loss; n=3), oligodendrocytes and neurons (brain autopsies, n=14) from Profs. Kirsty L. Spalding and Henrik Druid, Karolinska Institute, Stockholm, and research grade cadaveric pancreatic islets from Prof. James Shapiro, University of Alberta (n=16). In all cases tissues were obtained and transferred in compliance with local laws and after the approval of the local ethics committee on human experimentation. Sixteen cell types were obtained from commercial sources, including 15 from Lonza Walkersville, Walkersville, MD, U.S.A. and one from Sigma Aldrich. Three pancreatic islet preparations were obtained from the Integrated Islet Distribution Program (IIDP, https://iidp.coh.org).

Note that full information on the approval of the study protocol must also be provided in the manuscript.

Field-specific reporting

Please select the one below that is the best fit for your research. If you are not sure, read the appropriate sections before making your selection.

☒ Life sciences ☐ Behavioural & social sciences ☐ Ecological, evolutionary & environmental sciences

For a reference copy of the document with all sections, see nature.com/documents/nr-reporting-summary-flat.pdf

Life sciences study design

All studies must disclose on these points even when the disclosure is negative.

Sample size	Based on sample availibility
Data exclusions	Healthy samples, Low sequencing coverage
Replication	Yes, across individuals. We define a similarity score between two samples as the fraction of blocks containing ≥3 CpGs, and ≥10 binary observations (sequenced CpG sites), where the average methylation of the two samples differs by ≥0.5. Only cell types with n≥3 FACS-sorted replicates from different donors are considered (136 samples in total).
Randomization	N/A
Blinding	Not relevant

Reporting for specific materials, systems and methods

We require information from authors about some types of materials, experimental systems and methods used in many studies. Here, indicate whether each material, system or method listed is relevant to your study. If you are not sure if a list item applies to your research, read the appropriate section before selecting a response.

Materials & experimental systems

n/a	Involved in the study
☐	☒ Antibodies
☒	☐ Eukaryotic cell lines
☒	☐ Palaeontology and archaeology
☒	☐ Animals and other organisms
☒	☐ Clinical data
☒	☐ Dual use research of concern

Methods

n/a	Involved in the study
☒	☐ ChIP-seq
☐	☒ Flow cytometry
☒	☐ MRI-based neuroimaging

Antibodies

Antibodies used	Attached table S1
Validation	Described in attached file "Supplementary Information"

Flow Cytometry

Plots

Confirm that:

☒ The axis labels state the marker and fluorochrome used (e.g. CD4-FITC).

☒ The axis scales are clearly visible. Include numbers along axes only for bottom left plot of group (a 'group' is an analysis of identical markers).

☒ All plots are contour plots with outliers or pseudocolor plots.

☒ A numerical value for number of cells or percentage (with statistics) is provided.

Methodology

Sample preparation	Described in file "Supplementary Information"
Instrument	FACS BD Aria
Software	BD FACSDiva 8.0.1
Cell population abundance	Described in file "Supplementary Information"
Gating strategy	Described in file "Supplementary Information"

☒ Tick this box to confirm that a figure exemplifying the gating strategy is provided in the Supplementary Information.
